# Addressing MISEV guidance using targeted LC‐MS/MS: A method for the detection and quantification of extracellular vesicle‐enriched and contaminant protein markers from blood

**DOI:** 10.1002/jex2.56

**Published:** 2022-09-05

**Authors:** Lauren A. Newman, Zivile Useckaite, Andrew Rowland

**Affiliations:** ^1^ College of Medicine and Public Health Flinders University Adelaide South Australia

**Keywords:** extracellular vesicles, liquid chromatography tandem mass spectrometry, plasma, protein markers, sample characterization, serum

## Abstract

Extracellular vesicles (EVs) are membrane‐bound nanosized particles released by cells into bodily fluids containing an array of molecular cargo. Several characteristics, including stability and accessibility in biofluids such as blood and urine, make EVs and associated cargo attractive biomarkers and therapeutic tools. To promote robust characterisation of EV isolates, the minimal requirements for the study of extracellular vesicles (MISEV) guidelines recommend the analysis of proteins in EV samples, including positive EV‐associated markers and negative contaminant markers based on commonly co‐isolated components of the starting material. Western blot is conventionally used to address the guidelines; however, this approach is limited in terms of quantitation and throughput and requires larger volumes than typically available for patient samples. The increasing application of EVs as liquid biopsy in clinical contexts requires a high‐throughput multiplexed approach for analysis of protein markers from small volumes of starting material. Here, we document the development and validation of a targeted liquid chromatography tandem mass spectrometry (LC‐MS/MS) assay for the quantification of markers associated with EVs and non‐vesicle contaminants from human blood samples. The assay was highly sensitive, requiring only a fraction of the sample consumed for immunoblots, fully quantitative and high throughput. Application of the assay to EVs isolated by size exclusion chromatography (SEC) and precipitation revealed differences in yield, purity and recovery of subpopulations.

## 1 INTRODUCTION

In recent years, the field of extracellular vesicle (EV) research has exploded with publications documenting their biological properties, functionality, and potential diagnostic, prognostic and therapeutic applications in human disease (Shao et al., 2018; Théry et al., 2018; Van Deun et al., 2017). The term EV describes a heterogenous population of membrane‐bound vesicles; including small EVs, such as exosomes (50–150 nm), that arise from endosomal pathways within the cell, and EVs of various sizes up to 1000 nm in diameter, shed directly from the plasma membrane (microvesicles) (Kowal et al., 2016; Newman et al., 2020). Whilst originally perceived simply as a pathway for cellular garbage disposal, EVs are increasingly recognised for their roles in local and systemic intercellular communication, diverse physiological processes and disease progression (Newman et al., 2020; Shao et al., 2018). These functions are facilitated via the transfer of biologically active cargo, including nucleic acids, proteins and lipids, into recipient cells resulting in phenotypic and functional changes (Greening et al., 2017; Newman et al., 2020).

The stability conferred through encapsulation within the vesicle membrane and accessibility in biological fluids such as blood and urine, makes EV cargo attractive as biomarkers and therapeutic tools (Hirsova et al., 2016). However, their clinical application is hindered by several challenges, particularly with respect to the competing imperatives of recovery and purity of isolated preparations (Newman et al., 2020; Van Deun et al., 2017; Webber & Clayton, 2013). To promote the standardisation of EV methodologies and reporting, the International Society for Extracellular Vesicles (ISEV) has provided guidelines that set out the minimal requirements for the study of extracellular vesicles (MISEV) (Théry et al., 2018). Researchers must provide robust evidence to claim the presence of EVs in isolates and assign physiological properties or functions to them. In addition, the EV‐TRACK knowledgebase provides a platform for the detailed recording of experimental procedures through a checklist of 115 parameters, from which studies are assigned an EV‐METRIC reflecting the capacity for the experiments to be properly interpreted and reproduced (Van Deun et al., 2017).

A key component of the EV‐METRIC is analysing samples for the presence of accepted EV markers and absence or depletion of markers not associated with EVs (Van Deun et al., 2017) (±EV markers). EV enriched proteins are derived from the plasma membrane or cytosol and reflect the process of biogenesis and sorting of cargo (Shao et al., 2018). MISEV (2018) defines two categories of proteins to be analysed in all preparations, in order to robustly claim the presence of EVs (Van Deun et al., 2017). Markers frequently identified from Category 1 (transmembrane or GPI‐anchored proteins) include tetraspanins (CD9, CD81, CD63) and major histocompatibility complex class 1 (MHC1) (Kowal et al., 2016; Shao et al., 2018; Théry et al., 2018). Category 2 comprises proteins that are incorporated from the cytosol into EVs, largely due to lipid‐ or membrane protein‐binding capacity. Examples of these are tumour susceptibility gene 101 (TSG101), heat shock 70 kDa proteins and flotillins ‐1 & ‐2 (Van Deun et al., 2017). A third category of commonly co‐isolated contaminants is also given for assessing purity. These are selected with respect to EV source; for example, apolipoproteins or albumin in blood‐derived EV isolates (Théry et al., 2018). Further, proteins expressed in intracellular compartments other than plasma membrane or endosome, such as endoplasmic reticulum (ER) or nucleus, may be used as markers of large EVs, cellular components or apoptotic blebs (Shao et al., 2018). Common examples include calnexin, endoplasmin (GP96), or histones (Kowal et al., 2016; Théry et al., 2018; Webber & Clayton, 2013), and comprise Category 4, which must be addressed by researchers claiming the specific isolation of small EVs (Théry et al., 2018).

Currently, western blotting is the most common approach used to address reporting requirements relating to analysis of ±EV markers (Théry et al., 2018). While western blotting is an established method for protein detection, the approach has many inherent limitations that impact applicability to certain sample types. By way of example, as an antibody‐based (immunoblotting) method, western blotting is semi‐quantitative and can reliably detect only one analyte per sample. This may not be an issue when working with EVs isolated from cell culture media as sample volumes are plentiful, allowing for multiple parallel analyses, and there is a greater ratio of vesicles to particulate contamination (Kreimer et al., 2015; Van Deun et al., 2017). This does, however, become a key limitation in the context of addressing reporting requirements when working with biospecimens from clinical trials or patient cohorts, as the resulting EV sample volume is often insufficient to accommodate multiple western blot analyses as control experiments. Additionally, the biospecimen sample matrix is typically more complex and variable between samples, which can impact the quality of western blot analysis. Importantly, western blots can also be limited by the performance of antibodies, as non‐specific binding can increase background and reduce confidence in analyte detection (Liebler & Zimmerman, 2013). As EVs become an increasingly important “liquid biopsy” platform and their application to clinical biospecimens gains increasing attention, there is a need for a robust, higher throughput, multiplexed approach to address ±EV marker reporting requirements, ideally utilising the same platform that is applied to biomarkers of interest. Particularly when working with biospecimens, different EV isolation methods are known to enrich specific EV sub‐populations and differ in terms of EV recovery and the extent and composition of vesicular and non‐vesicular contamination. Accordingly, it is important to consider the compatibility of isolation strategy with the analytical platform.

In recent years liquid chromatography mass spectrometry (LC‐MS/MS) analyses have facilitated expansive proteomic profiling of EVs (Rosa‐Fernandes et al., 2017; Shao et al., 2018). LC‐MS/MS has been applied in both untargeted and targeted workflows, to qualitatively screen for the presence of proteins and to quantify the abundance of specific proteins, respectively. Targeted LC‐MS/MS based protein quantification typically involves the enzymatic digestion of proteins into peptides, separation by reverse phase liquid chromatography, and quantification of specific fragmentation patterns associated with the peptide of interest using a triple quadrupole (QQQ) mass spectrometer (Kreimer et al., 2015; Rosa‐Fernandes et al., 2017). This approach, referred to as multiple reaction monitoring (MRM), is highly sensitive, reproducible, and depending on instrument configuration can simultaneously analyse up to 20 proteins in a single sample. Additionally, targeted LC‐MS/MS analysis enables absolute analyte quantification in a complex matrix when the magnitude of the response for the endogenous analyte is normalised using a stable isotope labelled (SIL) peptide and compared to an external calibrator spiked into a comparable matrix at a known concentration (Greening et al., 2017; Kreimer et al., 2015).

Few studies have previously employed targeted LC‐MS/MS assays to assess purity of EVs from blood (Park et al., 2021; Wang et al., 2017), and have been useful in the development of novel isolation strategies (Nguyen et al., 2021) or to gain insight to membrane origin of circulating vesicles (Zhang et al., 2020). Of these studies, only that by Park et al. (2021) was performed on clinically relevant volumes of sample (100 μl plasma, while others used up to 200 ml). However, the proteins included in this panel covered cytosolic EV‐enriched proteins and non‐EV contaminants while transmembrane (MISEV category 1) proteins were notably absent. Thus, the present manuscript describes the development and validation of a novel MRM‐based panel specifically designed to address MISEV guidelines. This approach is sensitive, fully quantitative and high throughput. Establishing the presence of EV markers and depletion of contaminants is a critical component of sample characterisation, likely to be expanded upon in new iterations of MISEV (Witwer et al., 2021). Since the 2018 guidelines recognised the challenge of performing several characterisation experiments when sample volume is limited, we validate the application of this platform in small starting volumes. Hence, the method described here may be generalised to other EV‐based research applications, of different cell‐types or (patho)physiological condition, but we anticipate its particular value for the analysis of clinical biospecimens.

## 2 METHODS

### 2.1 Blood samples

Venous blood from healthy volunteers was collected into Z Serum Sep Clot Activator tubes or K_3_EDTA plasma vacuettes (Greiner Bio‐one, Frickenhausen, Germany) and centrifuged twice at 2500 g for 15 min at 10°C. Serum or plasma was extracted and stored at −20°C until analysis. The study was approved by the Southern Adelaide Clinical Human Research Ethics Committee (SAHREC; number 261.18). Serum and plasma samples from patients with non‐alcoholic fatty liver disease (NAFLD) were purchased from Discovery Life Sciences (Hunstville, AL, USA).

### 2.2 EV isolation

#### 2.2.1 qEV size exclusion chromatography

qEV Original (Legacy) 35 and 70 nm size exclusion chromatography (SEC) columns (iZon Science, Christchurch, NZ) were used to isolate EVs from serum or plasma. Prior to EV isolation, columns were equilibrated to room temperature (RT) and washed with 10 ml of 0.2 μm filtered phosphate‐buffered saline (PBS). Serum (500 μl) was loaded into the sample reservoir and allowed to completely pass into the column before PBS was added (no more than 2 ml at any time) to begin elution. The first six fractions (3 ml) eluted from the column was discarded and vesicles were collected as pooled fractions 7–11 (2.5 ml) into 5 ml Protein LoBind tubes (Eppendorf). Pooled vesicle fractions were mixed gently by inversion and concentrated to 100 μl using Amicon Ultra‐4 centrifuge filters (30 kDa, Millipore‐Sigma) pre‐conditioned with PBS. Concentrated vesicle isolates were stored at −80°C until analysis.

#### 2.2.2 ExoQuick precipitation

Serum was centrifuged for 15 min at 3000 × *g* at 10°C to remove debris. Spun serum (500 μl) was combined with ExoQuick™ precipitation solution (126 μl) and mixed eight to 10 times by gentle inversion. Samples were incubated for 30 min on ice then centrifuged for 30 min at 1500 g at 4°C to pellet EVs and again for 5 min in the same conditions, each time aspirating all supernatant. The pellets were resuspended in 100 μl of filtered PBS/RIPA buffer and stored at −80°C until analysis.

### 2.3 Nanoparticle tracking analysis

Nanoparticle tracking analysis (NTA) was performed to quantify particle concentration and size distribution in EV samples using a NanoSight NS300 (Malvern Analytical, UK). Samples were diluted between 1:500 and 1:20000 in freshly 0.2 μm filtered PBS. Five 60‐s videos were captured at camera level 14 with a continuous sample flow (flow rate 100). Videos were analysed at detection threshold five using NTA 3.0 software.

### 2.4 Transmission electron microscopy

Samples were prepared based on a previously published protocol (Newman et al., 2021). Briefly, Ted‐Pella B 300 M carbon‐coated grids (Ted‐Pella, Redding, CA, USA) were cleaned and hydrophilized using plasma glow discharge for 15 seconds (Gatan SOLARUS Advanced Plasma Cleaning System, Gatan, Inc., Pleasanton, CA, USA) prior to use. Five microlitres of sample in 0.2 μm‐filtered PBS was placed on carbon‐coated grids for 5 min. Carbon grids were washed once (15 s) at RT with 0.2 μm filtered PBS and were contrasted with 2% uranyl acetate (3 min, RT), washed once, and examined by FEI TECNAI Spirit G2 TEM (Thermo Fisher Scientific, Waltham, MA, USA) operated at 100 kV. TEM images were acquired at magnifications of 49,000× and 68,000× (Figure S1).

### 2.5 Human liver microsome preparation

Pooled human liver microsomes (HLMs) were prepared by differential centrifugation as described by Bowalgaha et al. (2005). Briefly, liver portions (<1 cm thickness) were suspended in phosphate buffer (0.1 M, pH 7.4) containing potassium chloride (KCl; 1.15% w/v) and minced using scissors. The minced liver tissue was homogenized, initially with a Janke and Kunkle Ultra Turax at a speed of 24000 rpm, and then with a Potter‐Elvehem homogenizer (driven by a power drill) at a speed of 1480 rpm. The homogenized tissue was centrifuged at 3000 g for 10 min at 4°C, and again at 10,000 g for 10 min at 4°C. The supernatant layer was collected and centrifuged at 105,000 g for 1 h at 4°C. The resulting pellet was re‐suspended in phosphate buffer (0.1 M, pH 7.4) containing KCl (1.15% w/v) and then centrifuged at 105,000 g for 1 h at 4°C. The final microsomal pellet was suspended in phosphate buffer (0.1 M, pH 7.4) containing glycerol (20% v/v), aliquoted into 400 μl samples, and stored at –80°C until use. Equal protein amounts of microsomes from five human livers (H7, female 44 years old [y/o]; H10, female 67 y/o; H12, male 66y/o; H29, male 45y/o; and H40, female 54y/o) were used for the purpose of this study. Approval for the use of human liver tissue in xenobiotic metabolism studies was obtained from both the Clinical Investigation Committee of Flinders Medical Centre and from the donors’ next of kin. All livers were obtained within 60 min of death and were immediately sliced and frozen in liquid nitrogen. Once frozen, livers were stored at ‐80°C until use.

### 2.6 Protein isolation from EVs for immunoblots

EVs isolated by qEV were lysed by mixing an equal volume (6 μl) of EV sample in PBS with ice‐cold RIPA (Radioimmunoprecipitation assay) lysis buffer (Thermo Fisher Scientific, IL, USA). ExoQuick isolated EVs (pellet) was lysed in 100 μl of the above RIPA lysis buffer. All samples were incubated on ice for 25 min, centrifuged at 10,000 g for 10 min at 4°C. Soluble protein was measured by micro BCA assay (Thermo Fisher Scientific, IL, USA), according to manufacturer's instructions. Briefly, working reagent (WR) was prepared using MA:MB:MC at 25:24:1 ratio. Lysed samples were diluted up to 300 μl in 0.2 μm‐filtered PBS. In a 96‐well plate, equal volume of WR and either sample or bovine serum albumin (BSA) standard were mixed and assayed in duplicate. Absorbance of samples at 562 nm were compared to that of BSA standard curve (0–200 μg/ml) to determine protein concentration, using a SuperMax plate reader (Molecular Devices, CA, USA).

### 2.7 Protein isolation from HLM and serum for immunoblots

HLM and serum protein was isolated by mixing equal volumes of HLM and ice‐cold RIPA buffer, incubated on ice for 25 min, centrifuged at 10,000 g for 10 min at 4°C. Soluble protein was measured, as above, by micro BCA assay (Thermo Fisher Scientific, IL, USA).

### 2.8 Immunoblotting

EV, HLM, and serum protein (35 μg), isolated as described above, was used for immunoblotting as we previously described (Useckaite et al., 2020), except that 5% BSA/TBS containing 0.1% Tween 20 (TBST) was used. Protein lysates (EV, HLM, and serum) were resolved on gradient SDS gels (Bio‐Rad Laboratories, CA, USA) and the proteins where then transferred to Immun‐Blot LF polyvinylidene difluoride (PVDF) membrane, 0.45 lm (Bio‐Rad Laboratories, CA, USA), using a Turbo Blot transfer unit (Bio‐Rad Laboratories, CA, USA). Stain‐free imaging of the gel was performed using a ChemiDoc MP imager (Bio‐Rad Laboratories, CA, USA) with a 1‐min stain activation time as previously described (Colella et al., 2012). Total protein images were obtained at pre‐blocking of PVDF (Figure S2). PVDF membranes were blocked with 5% (w/v) BSA in TBST and incubated overnight at 4°C with primary antibodies.

Primary antibodies (in 5%BSA/TBST) from Abcam (Abcam, Cambridge, MA, USA) were anti‐human CD9 (Cat.#:ab92959; 1/1000); anti‐human CD63 (Cat.#:ab68418; 1/1000); anti‐human CD81 (Cat.#:ab109201; 1/1000); and anti‐human Calnexin (Cat.#: ab2791; 1/1000). Primary antibody for anti‐human TSG101 was from Invitrogen (Thermo Fishes Scientific, IL, USA; Cat.#:PA5‐31260; 1/1000). Secondaries from Cell Signalling Cell Signalling Technology, MA, USA) were anti‐mouse IgG, HRP‐linked (Cat.#:7076; 1/1000) or anti‐rabbit IgG, HRP‐linked (Cat.#:7‐74; 1/1000). HLM‐lysate was included in all gels as a positive control. Serum lysate was added as a positive control for Albumin.

SuperSignal West Femto Chemiluminescent Substrate Kit (Thermo Fisher Scientific, IL, USA) was used for detection, imaging was performed using an automated ChemiDoc Touch Imaging System (Bio‐Rad Laboratories, CA, USA) and densitometric analysis was performed using ImageJ tool (https://imagej.nih.gov/ij/index.html).

### 2.9 Peptide digestion

EVs isolated by SEC columns and by ExoQuick precipitation, 50 and 10 μl, respectively, were diluted up to 100 μl in PBS; containing between 70 and 20 μg of SEC EV protein and 1132–1683 μg of ExoQuick EV protein. EVs were lysed by vortexing for 10 min using a MixMate sample mixer (Eppendorf) followed by three freeze‐thaw cycles. Lysed samples were mixed with 50 μl of ammonium bicarbonate (pH 7.8) and incubated with dithiothreitol (12.5 mM) for 90 min at 60°C. Samples were cooled to RT prior to addition of iodoacetamide (23.5 mM) and incubation for 60 min at 37°C. Trypsin Gold was then added to protein samples in a ratio of 1:40 and incubated for 17 h at 37°C. Samples were mixed with 20 μl of formic acid (10% v/v) in order to terminate digestions, then centrifuged at 16,000 g for 10 min at 4°C. Resulting supernatant (100 μl) was spiked with a SIL peptide cocktail (final [nM]: ALB: 10; CD81: 0.4; CD9: 0.1; CANX, TSG101: 0.2). SIL peptides were obtained from Vivitide (MA, USA), all of isotopic purity >99%. Digested samples containing SIL peptides were run immediately and stored in the autosampler at 15°C over the course of the run. A 5 μl aliquot of digested protein was injected for analysis by LC‐MS/MS (Table S1). HLM and serum, diluted 1:100 in PBS, were digested in the same conditions and run as positive controls.

### 2.10 Chromatography

Chromatographic separation of analytes was performed on an Agilent Advance Bio Peptide Map column (100 mm × 2.1 mm, 2.7 μm) using an Agilent 1290 Infinity II liquid chromatography system. The temperature of the sample and column compartments was maintained at 15°C and 30°C, respectively. A panel of analytes comprising the EV makers CD81, CD9, and TSG101, and contaminants calnexin (CANX) and albumin (ALB), were separated by gradient elution with a flow rate of 0.2 ml/min. The mobile phase consisted of 0.1% formic acid in water (mobile phase A) and 0.1% formic acid in acetonitrile (mobile phase B) held in a proportion of 97% A and 3% B for the first 3 min. The proportion of mobile phase B was then increased linearly to 30% over 30 min then increased to 50% in 5 min and held for 1 min before returning to 3% over a further 5 min. Lastly, mobile phase B was held at 3% to re‐equilibrate the column for 5 min.

### 2.11 Mass spectrometry

Column eluant was monitored by mass spectrometry using an Agilent 6495B QQQ mass spectrometer operating in positive electron spray (ESI+) mode. Target proteins were included in the panel in accordance with MISEV reporting guidelines. Proteotypic peptides for each protein marker were screened in EV samples and/or positive controls (HLM) and confirmed using Skyline software. Peptides contained between seven and 22 amino acids for uniqueness and mass range of QQQ instrument. Peptides had no methionine or cysteine residues. Sites of mutagenesis or post‐translational modifications were avoided. For one peptide per protein, one quantifier and two qualifier ion transitions were included for optimisation of the MRM method based on signal intensity (Table 1). Three types of each analyte were detected; synthetic isotope labelled (SIL), endogenous and synthetic light peptide; as the latter was spiked into samples to supplement endogenous levels where required for assay validation. Skyline software was used to verify transitions and to select the optimal collision energy for each transition from seven predicted voltages. MassHunter Optimiser Software was used to optimise source parameters: capillary voltage, nebuliser pressure and nozzle voltage; and cell accelerator voltage was optimised manually between 3 and 8 V. Identities of endogenous peptides were confirmed by comparing retention time and quantifier/qualifier transition ratios to respective SIL peptide standards.

**TABLE 1:** Analyte sequences and transitions used for multiple reaction monitoring

Analyte	Type	Sequence	Retention time (min)	Precursor Ion	Product Ions			Collision energy (eV)
ALB	SIL	H2N‐LVNEVTEFA **K^**‐OH	20.7	579.3	603.3 (y5+)	702.4 (y6+)	**945.5 (y8+)**	18.8
	Light	H2N‐LVNEVTEFAK‐OH	20.7	575.3	595.3 (y5+)	694.4 (y6+)	**937.5 (y8+)**	18.8
CD81	SIL	H2N‐QFYDQALQQAVVDDDANNA **K^**‐OH	23.9	754.4	870.4 (y8+)	**969.4 (y9+)**	1068.5 (y10+)	17.3
	Light	H2N‐QFYDQALQQAVVDDDANNAK‐OH	23.9	751.7	862.4 (y8+)	**961.4 (y9+)**	1060.5 (y10+)	17.3
CD9	SIL	H2N‐DVLETFTV **K^**‐OH	24.3	530.3	603.4 (y5+)	732.4 (y6+)	**846.5 (y7+)**	22.3
	Light	H2N‐DVLETFTVK‐OH	24.3	526.3	595.4 (y5+)	724.4 (y6+)	**837.5 (y7+)**	22.3
CANX	SIL	H2N‐IVDDWANDGWGL **K^**‐OH	27.2	748.9	797.4 (y7+)	**868.4 (y8+)**	1054.5 (y9+)	24.1
	Light	H2N‐IVDDWANDGWGLK‐OH	27.2	744.9	789.4 (y7+)	**860.4 (y8+)**	1046.5 (y9+)	24.1
TSG101	SIL	H2N‐GVIDLDVFL **K^**‐OH	32.5	563.8	742.5 (y6+)	**857.5 (y7+)**	970.6 (y8+)	18.4
	Light	H2N‐GVIDLDVFLK‐OH	32.5	559.8	734.4 (y6+)	**849.5 (y7+)**	962.6 (y8+)	18.4

*Note*: SIL (^): Stable isotope labelled peptide; bold and underlined letter = heavy labelled amino acid. Bold values indicate product ions used as quantifier transitions.

### 2.12 Assay validation and calibration

Calibration standards (*n* = 8) were prepared to span the concentration ranges associated with qEV70 EV isolates, and to ensure a robust minimal concentration to exclude contamination (CANX and ALB) from human serum. In this range, assay linearity was determined for each analyte according to linear regression analysis. Assay sensitivity was determined for the panel. The limit of detection (LOD) was defined as a signal to noise ratio of 3:1 and the lower limit of quantification (LLOQ) as 5× the LOD.

Precision was assessed on the basis of intra‐ and inter‐day variability in the slope produced by calibration curves run in triplicate on three separate days. Variability was recorded as percent relative standard deviation (% RSD) of triplicate injections (within run) and of average slope across different days (between runs). Repeatability was assessed by five consecutive injections of a mid QC sample and variability recorded as % RSD. Accuracy was determined based on the recovery of SIL peptide spiked into quality control (QC) samples at low (ALB: 6; CD81: 0.6; CD9: 0.06; CANX, TSG101: 0.12 [nM]), mid (ALB: 10; CD81: 1; CD9: 0.1; CANX, TSG101: 0.2 [nM]) and high (ALB: 18; CD81: 1.8; CD9: 0.18; CANX, TSG101: 0.36 [nM]) concentrations within the calibration curve. Carryover was assessed in two consecutive blank injections following the highest calibration standard.

The stability of analytes was evaluated in duplicate EV samples. Samples were kept at −20°C, 4°C, or 15°C and analysed at baseline and after 6, 24, and 48 h. Concentration was determined at each time point and changes from baseline of less than 20% were accepted as stable.

Matrix effects were assessed based on absolute and relative recovery of SIL peptides spiked in EV matrix or mobile phase. Calibrators 1 and 6 and a middle QC sample were prepared and analysed in each matrix and used to generate curves. Matrix effects were reported as % difference in slope and precision in each matrix was based on triplicate injections of each QC.

The reproducibility of the protocol was assessed based on the reproducibility of detecting analytes and of quantifying analytes. EVs were isolated in triplicate from the serum of three donors by each of the three isolation methods as described above, and peptide digests were performed in duplicate, as also previously described. Reproducible detection across replicate isolations was defined by samples with average normalised response > LOD and reproducible detection in peptide digests was defined by equivalent response (both duplicates are < or > LOD) in each pair.

Additional analyses were performed to demonstrate the generalizability of the assay. Specifically, these analyses demonstrate the capacity to detect EV markers in plasma from healthy controls and serum and plasma from subjects with NAFLD. These analyses were performed using EVs isolated by two distinct isolation approaches.

### 2.13 Statistical analysis

Statistical analyses were performed using GraphPad Prism software version 9 (San Diego, CA, USA). Comparisons of group means were assessed by repeated measures one‐way ANOVA with Tukey test for multiple comparisons. Linear regression analysis was performed using Microsoft Excel version 16.

### 2.14 EV‐TRACK

We have submitted all relevant data of our experiments to the EV‐TRACK knowledgebase (EV‐TRACK ID: EV220163) (Van Deun et al., 2017).

## 3 RESULTS

### 3.1 Characterisation of EVs

**FIGURE 1:**
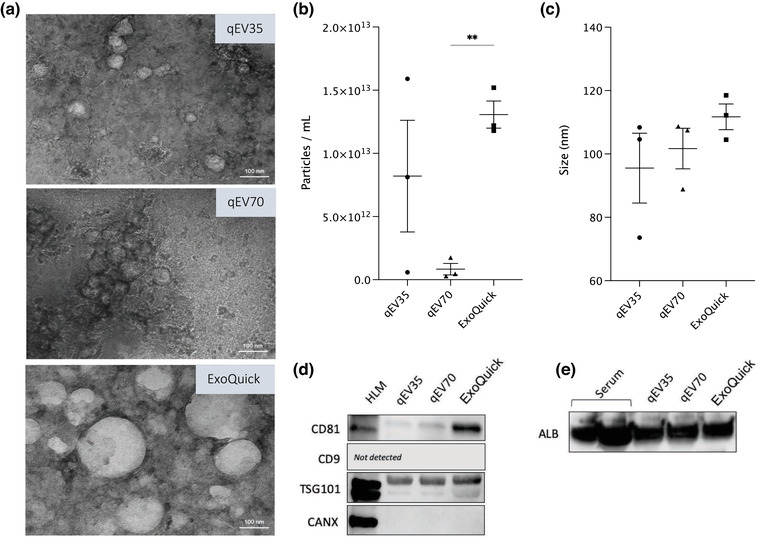
Characterisation of extracellular vesicles isolated by SEC and precipitation. (a) Transmission electron microscopy images. Direct magnification ×30,000. Scale bar = 100 nm. (b) Concentration and (c) Mean size of particles measured by nanoparticle tracking analysis. Data shown as mean ± standard deviation; **p < 0.01; (*n* = 3), One‐way ANOVA and Tukey test for multiple comparisons. (d) Representative images of key proteins considered to be markers of EVs (CD81, CD9, and TSG101), according to MISEV 2018 guidelines, in EVs isolated by qEV35, qEV70, and ExoQuick against HLM (non‐EV control). Endoplasmic reticulum protein, CANX, was included as a marker of cellular structures considered to be enriched in cells relative to EVs (i.e., non‐EV component, negative EV marker). (e) Representative image showing albumin contamination in EV samples isolated by qEV35, qEV70, and ExoQuick. Thirty‐five micrograms of lysed EV or serum protein loaded per lane. Two lanes of albumin represent 35 μg of protein that leaked to the neighbouring well due to pipetting error

EVs were isolated from human serum (*n* = 3) by each of three commercially available methods; qEV70 and qEV35 SEC columns, and ExoQuick precipitation. TEM images revealed characteristic morphology and structurally intact vesicles isolated by each method, however, high background from non‐vesicular contamination was prevalent in the ExoQuick image (Figure 1a). The mean particle concentration varied between methods, with significantly more particles isolated by ExoQuick compared to qEV70 (Figure 1b). Mean particle size measured by NTA was consistent across the three isolation methods (Figure 1c), although TEM images demonstrated the presence of a sub‐population of larger vesicles in the ExoQuick isolate.

Irrespective of EV isolation method, common EV markers CD81 and TSG101 were detected by immunoblots in all EV samples (Figure 1d and Figure S2–S4), although differences in apparent abundance were observed (Figure S6). TSG101 abundance was comparable between EVs isolated by each of the SEC columns, while a greater amount was detected in ExoQuick samples. Similarly, samples isolated by SEC columns displayed low levels of CD81, with the highest signal detected in ExoQuick‐isolated samples. Two bands were observed for TSG101 protein, in line with other publications using the same antibody (Hofmann et al., 2020; Schroeder et al., 2020). CD9 expression was not detected in any of the samples. The ER protein, CANX, was included in the analysis as a marker of non‐EV cellular structures (EV negative marker). CANX expression was below the LOD of the immunoblot method. While this method cannot provide a quantitative assessment, the lack of CANX detection suggests minimal contamination with cellular debris.

Since EVs were isolated from human serum, an additional immunoblot was run to compare albumin contamination across EV samples resulting from different methods of EV isolation (Figure 1e and S5). Lysed serum protein was analysed as a positive control. Similar size bands were observed at approximately 70 kDa across all EV samples, irrespective of isolation method. Importantly, blots were loaded with equivalent amount protein (35 μg) for each sample type, so they do not reflect the analyte abundance in equal volumes of starting material. Specifically, the volumes of serum corresponding to amount of loaded protein for EV samples isolated by qEV35, qEV70 and ExoQuick are 103, 115, and 1.9 μl, respectively (Table S1). This consideration of co‐isolated contaminants per serum volume highlights the vastly greater levels of albumin recovered in ExoQuick isolates compared to SEC. Indeed, SEC has been reported to isolate a greater ratio of vesicle to serum proteins, compared to precipitation reagents or conventional techniques such as ultracentrifugation, and can reproducibly isolate vesicles containing characteristic EV markers (Monguió‐Tortajada et al., 2019; Vanderboom et al., 2021). As qEV35 enriches for vesicles 35–350 nm in diameter while qEV70 enriches those 70–1000 nm, the former is prone to greater co‐isolation of lipoproteins from the blood. For these reasons, development of the peptide assay was primarily performed using vesicles isolated by qEV70.

### 3.2 MRM method development

Target proteins were selected to address the category reporting requirements outlined in the MISEV guidelines for EVs derived from blood products (serum or plasma) (Théry et al., 2018). The final protein panel comprised CD81 (Category 1[a]), CD9 (Category 1[b]) and TSG101 (Category 2a) as positive vesicle markers, albumin (ALB; Category 3) to represent matrix‐associated contamination in EV isolates from the blood, and calnexin (CANX; Category 4) as a marker of cellular debris or large vesicles. Selected tryptic peptides corresponding to each target protein were detected in vesicles isolates and/or positive controls (HLM) and one precursor ion with three product ion transitions of greatest intensities were included for optimisation (Table 1). Instrument settings were optimised as described in materials and methods and values for optimised parameters are given in Table 2. Chromatograms of SIL peptides spiked into digested EVs (qEV70) is shown in Figure S7. Retention times were highly reproducible for each analyte measured in calibration standards with RSD between 0.04% and 0.07 %.

**TABLE 2:** Mass spectrometer instrument settings

Parameter	Setting			
Time segment (min)	0–22	22–26	26–30	30–48
Delta EMV (V)	300	300	300	300
Capillary voltage (V)	3000	2500	3000	3000
Nebulizer pressure (psi)	30	25	30	30
Nozzle voltage (V)	1000	500	500	500
Cell accelerator voltage	5	3	5	4

### 3.3 Assay validation

#### 3.3.1 Linearity and sensitivity

**FIGURE 2:**
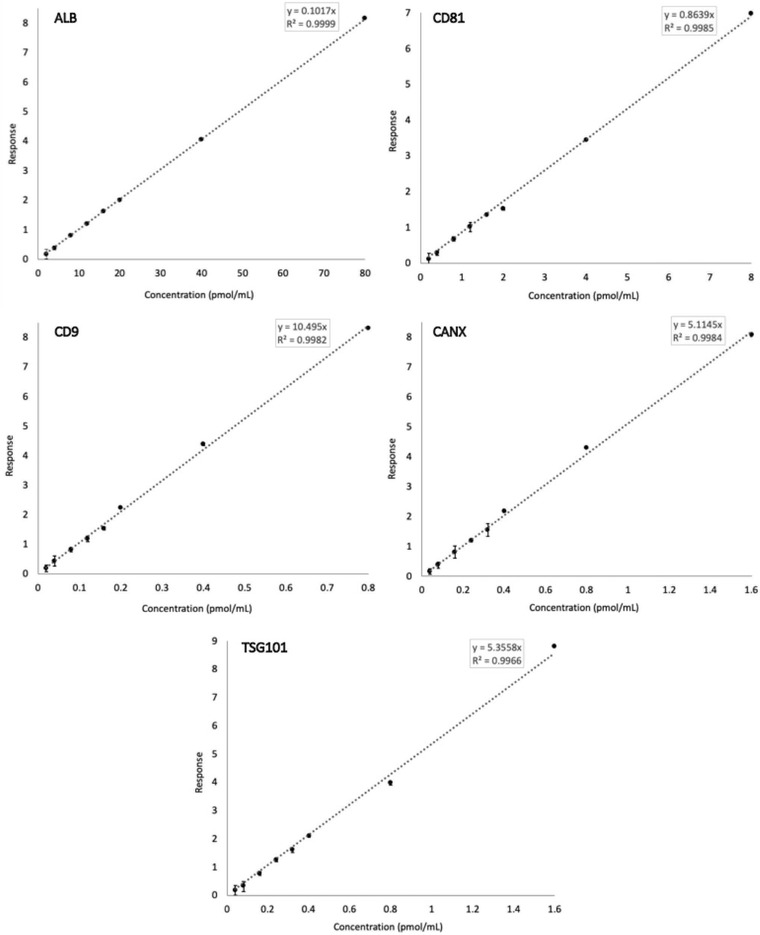
Calibration curves for the five analytes in the EV Marker Panel, produced by linear regression analysis. Error bars represent % relative standard deviation (% RSD) (*n* = 3)

Calibration curves were generated for each analyte in the panel to encompass the concentration range typically observed for normal human serum (calibrators 1–6) and at points two and four times beyond that, as may be observed with increased levels of circulating EVs in various disease states (calibrators 7 and 8) (Nguyen et al., 2021; Povero et al., 2020; Sehrawat et al., 2021). The required range varied considerably between positive and negative EV markers: Albumin was validated between 2.0 and 80 pmol/ml while this range was 100‐fold less for CD9 (Table 3). Linearity of response was assessed by linear regression analysis and produced coefficient of determination (*r* ^2^) values for each analyte ranging between 0.9966 and 0.9999 (Figure 2). The sensitivity of the assay was determined with respect to LOD and LLOQ, calculated as described in materials and methods. For most of the analytes, the validated calibration range extended towards the lower end of the assay's sensitivity. Details of these characteristics are summarised in Table 3.

**TABLE 3:** Details of calibration and quantification for analytes in the EV marker panel

Analyte	Calibration range (pmol/ml)	Calibration curve coefficient of determination (*r* ^2^)	Limit of detection (LOD) (pmol/ml)	Lower limit of quantification (LLOQ) (pmol/ml)
ALB	2.0–80	0.9999	0.004	0.020
CD81	0.2–8.0	0.9985	0.050	0.230
CD9	0.02–0.8	0.9982	0.005	0.025
CANX	0.04–1.6	0.9984	0.007	0.035
TSG101	0.04–1.6	0.9966	0.006	0.031

#### 3.3.2 Assay precision, repeatability and accuracy

Assay precision was assessed based on intra‐ and inter‐day variability in the slope of calibration curves run in triplicate on three separate days. The %RSD ranged from 0.4% to 13% for within‐run variability in analyte response, and between runs, variability ranged from 0.4% to 8% (Table 4). Instrument repeatability was also determined in five consecutive injections of the same sample and gave % RSD < 9 % for all analytes. Relative accuracy (within matrix) of the assay was determined by measurement of low, mid and high QC samples. These QC points were selected within the concentration range typically observed for normal human serum (calibrators 1–6); nominal concentrations are given in Table 5. For all analytes, accuracy ranged from 92% to 112% at low, 97%–116 % at mid, and 94%–110 % at high QC concentration (Table 5).

**TABLE 4:** Assay precision

	Variability (% RSD)	
Analyte	Intra‐day	Inter‐day
ALB	0.4	0.4
CD81	5.6	7.6
CD9	3.0	6.2
CANX	4.5	3.9
TSG101	13	4.6

*Note*: Relative standard deviation (% RSD) of slopes of triplicate calibration curves run within day and on three different days.

**TABLE 5:** Assay accuracy

	QC Concentration (pmol/ml)								
Analyte	Low			Mid			High		
	Nominal	Measured	% True	Nominal	Measured	% True	Nominal	Measured	% True
ALB	6.0	6.75	112	10	10.9	107	18	20.2	110
CD81	0.6	0.550	92	1.0	0.972	97	1.8	1.72	96
CD9	0.06	0.063	105	0.1	0.094	94	0.18	0.187	104
CANX	0.12	0.112	93	0.2	0.232	116	0.36	0.340	94
TSG101	0.12	0.120	100	0.2	0.212	106	0.36	0.392	109

#### 3.3.3 Carryover

Carryover was assessed in two consecutive "blank" injections of mobile phase following injection of the highest calibration standard. Blank injections involved a full LC injection cycle. Albumin response was detectable in both injections (injection 1 = 4× LOD; injection 2 = LOD) but did not reach the LLOQ. For all other analytes, peak area response was less than LOD in both first and second injections.

#### 3.3.4 Short‐term stability

**FIGURE 3:**
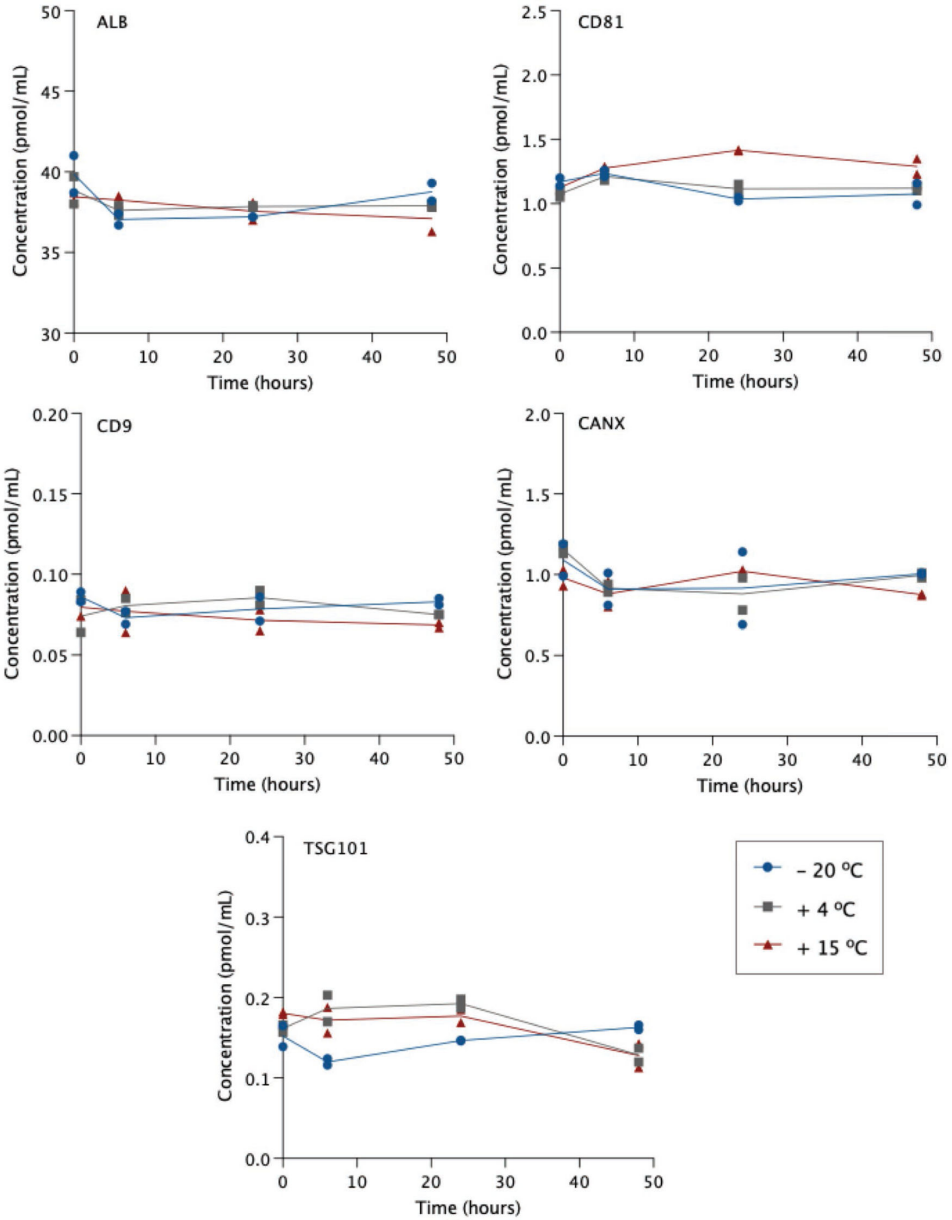
Short‐term stability of peptide concentration at different storage temperature

Analytes were tested for short term stability with storage at −20°C, 4°C, or 15°C. Concentration was determined at baseline and monitored in duplicate samples at 6, 24, and 48 h. To calculate concentration, peak area response for endogenous analytes (or light peptide supplemented where required) was normalised to known concentrations of respective SIL peptide spiked in at baseline. Concentration of all analytes was generally stable across each time point compared to baseline measures (Figure 3). ALB concentration varied less than 7% in all cases and variability between duplicates remained low over time (<4% RSD). Indeed, there were no notable trends in %RSD as most measures varied no more than 19% between duplicates; except for CANX, for which variability increased up to the 24 h mark, especially in samples kept at −20°C (up to 34%). CANX concentration was otherwise stable over time, with most variability ± 17% from baseline. CD81 concentration was stable in samples stored at −20°C and 4°C with <13% change from baseline. In 15°C samples, a slight decrease in SIL peptide response led to overestimated concentration by 26% at 24 h. Since SIL was spiked in at baseline and monitored concurrently with the light analyte, different rates of degradation will affect calculations of analyte concentration. When light and SIL peptide responses were assessed individually, there were also slight reductions in ALB and CD9 for samples kept at 15°C, but since signal was reduced in each light and SIL to a similar extent, no effect was observed in concentration. Lastly, TSG101 concentration was particularly stable over time in samples kept at −20°C and in 4°C and 15°C samples for 24 h. In the latter two, however, levels had decreased by 20% and 29%, respectively by 48 h. Overall, these data indicate that peptide concentration is stable during storage at −20°C and can also remain in the autosampler over the typical course of assay runs without significantly affecting results.

#### 3.3.5 Matrix effects

It is widely established that EV isolation methods are not equivalent in terms of both vesicle recovery and purity. The presence of non‐vesicular contaminants can impact downstream analyses including detection and quantification of EV cargo by LC‐MS/MS Thus, the effect of different EV matrices was assessed based on absolute and relative recovery of SIL peptides spiked into EV matrix or mobile phase. Relative recovery was determined in qEV35 and ExoQuick matrix using qEV70 as a comparator. Standard curves were generated using calibrators 1 and 6 and a middle QC. *R* ^2^ values exceeded 0.99 for all analytes; with the exception of CD81 in ExoQuick, which gave *r* ^2^ = 0.977 (Table 6). The ExoQuick CD81 curve was affected by imprecision at the lower calibration points. Precision is reported as %RSD of triplicate injections at each concentration level. The results were comparable with that observed in qEV70 matrix, though greater variability in CD81 quantification was observed in ExoQuick samples at the lower calibration point (27%RSD). Only 22% of the CD81 response was recovered in ExoQuick matrix compared to qEV70, these data indicate that this analyte is impacted in ExoQuick matrix such that limits of detection and quantification occur at higher concentrations. Similarly, ALB response in ExoQuick was 40% of that in qEV70. The response for the remaining analytes were greater in alternate matrices compared to qEV70, most notably TSG101 was 164% and 171% in qEV35 and ExoQuick, respectively. Hence, this analyte, along with CD9 and CANX, may be detected and quantified at lower concentrations in qEV35 and ExoQuick EVs.

**TABLE 6:** Relative recovery of SIL peptides in EV matrices compared to qEV70

	qEV35				
	Relative recovery (%)		Precision (%RSD)		
Analyte	*r* ^2^	% of curve in qEV70	Lower	Mid	Upper
ALB	0.9999	107	16	6.9	10
CD81	0.9955	91	18	13	7.1
CD9	0.9994	147	18	3.6	9.1
CANX	0.9993	128	3.3	8.6	2.3
TSG101	0.9909	164	14	3.5	2.2
Analyte	ExoQuick				

	Relative recovery (%)		Precision (%RSD)		
	*r* ^2^	% of curve in qEV70	Lower	Mid	Upper
ALB	0.9973	40	7.4	6.2	2.5
CD81	0.9770	22	27	15	14
CD9	0.9980	133	9.1	6.1	2.9
CANX	0.9998	127	2.3	4.3	0.9
TSG101	0.9974	171	10	3.6	1.7

To determine absolute recovery, the slope of the SIL peptide standard curves in each EV matrix were compared to that in mobile phase. Across each analyte, recovery in qEV70 matrix was observed at 38%–69% of mobile phase, with TSG101 most impacted (Table 7). On average, qEV35 matrix exhibited the least impact on analytes (absolute recovery 52%–88%). In ExoQuick matrix, albumin and CD81 peptide signals were significantly suppressed with recovery of just 22% and 13%, respectively.

**TABLE 7:** Absolute recovery of SIL peptides in EV matrices

	Recovery (% of curve in mobile phase)		
Analyte	qEV70	qEV35	ExoQuick
ALB	55	59	22
CD81	58	52	13
CD9	57	84	76
CANX	69	88	87
TSG101	38	54	57

The generalisability of the assay is demonstrated by analyses in serum and plasma from healthy donors and subjects with NAFLD. Analysis of markers in EVs isolated from plasma of NAFLD patients (*n* = 4) and healthy controls (*n* = 5) by qEV70 demonstrated a comparable capacity (relative to healthy serum) to detect positive EV markers and albumin. However, calnexin was only detected in 20% of healthy plasma EV samples. In EVs isolated by ExoQuick from NAFLD patient serum, markers were consistently detected across samples (Table S2).

#### 3.3.6 Reproducibility of analyte detection and quantification

The reproducibility of the protocol was evaluated using the SEC and precipitation‐based EV isolation methods. EVs were isolated from human serum (*n* = 3) in triplicate using each of the three methods and duplicate peptide digests were analysed on the panel (i.e., 18 per isolation method). Concentration of analytes was determined based on normalised response (endogenous/SIL) in isolates from equivalent volumes of starting material using the three methods of isolation. Reproducibility was assessed based on analyte detection and analyte quantification, using LOD and LLOQ adjusted to reflect the observed effects of alternate EV matrices on relative recovery, as described above.

**FIGURE 4:**
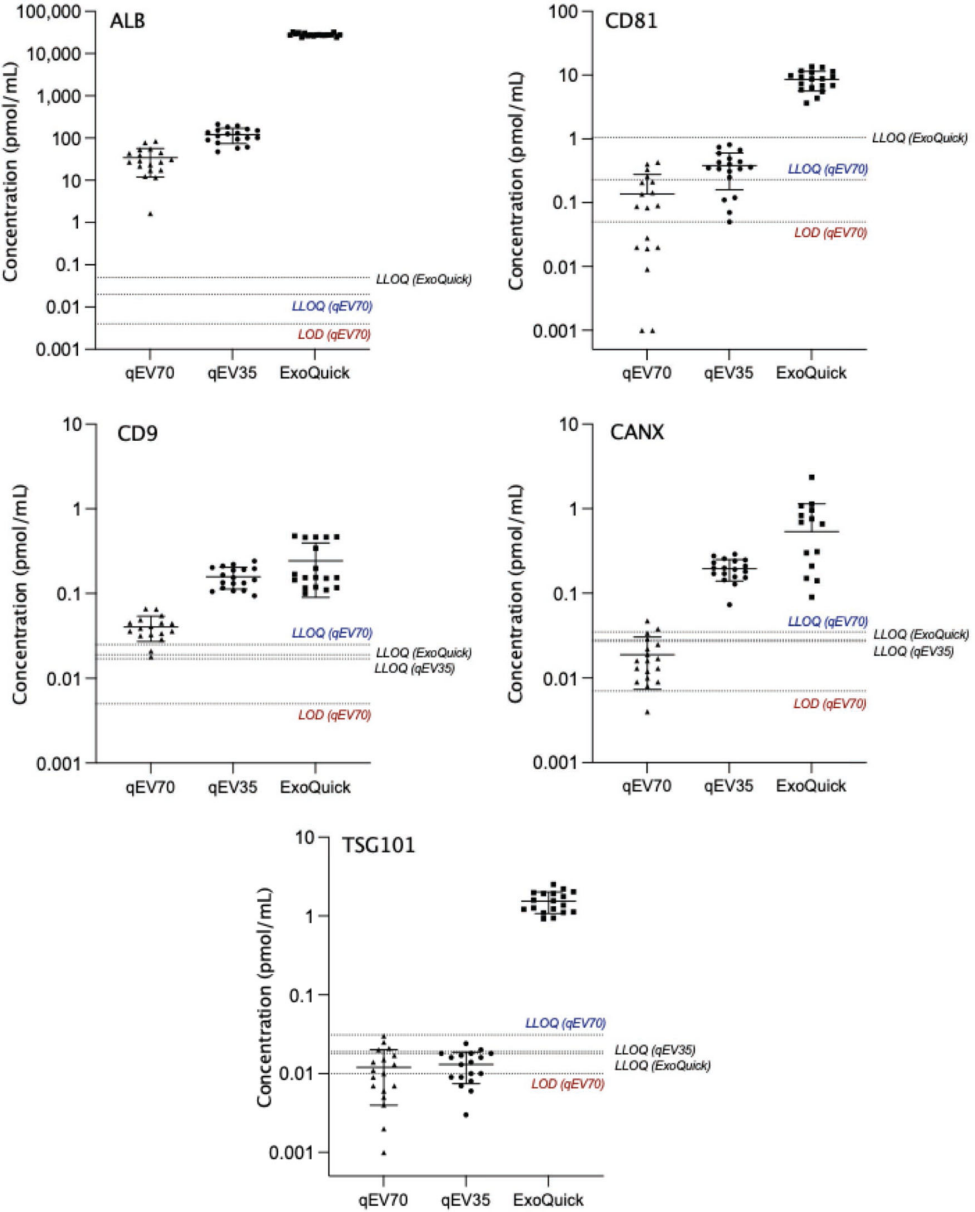
Mean (±SD) concentration of analytes measured in duplicate peptide digests from triplicate EV isolations using different methods (*n* = 3). Lower limit of quantification (LLOQ) and limit of detection (LOD) are indicated for qEV70 and LLOQ is given for analytes in alternate matrices where matrix effects (relative recovery differs >20%) were observed

Given the primary function of the EV Marker Panel is to demonstrate the presence or absence of positive and negative markers in accordance with the MISEV guidelines, we first sought to determine the reproducibility of analyte detection in replicate samples. Reproducibility of isolation was based on average normalised response > LOD across triplicate isolations, and reproducibility of peptide digests was defined by equivalent response (both duplicates are < or > LOD) in each pair (Table 8). Albumin and CD9 was detected in 100% of isolations and digests from all isolation methods. CD81 was also detectable in 100% of isolations and digests from qEV35 and ExoQuick EVs. However, from two of the donors, CD81 was not detected in qEV70 EVs in one isolation each, which reduced isolation reproducibility to 78% overall for this analyte. Further, some discrepancy between duplicate digests was observed, which may be attributed to the low abundance of CD81 in these samples. CANX and TSG101 were also detected reproducibly across isolations and digests in each isolation method. To expand the applications of the assay beyond the binary determination of analyte detection, to those such as quantifying marker abundance for use as a normalisation strategy, the assay must demonstrate reproducibility of quantification. To this end, variability in concentration was determined in triplicate isolations and duplicate digests and presented as %RSD (Table 9). The variability of digests was less than 20% for all analytes above LLOQ in each isolation method. Cases in which replicate values were < LLOQ were considered reproducible, since the effect of noise precludes the calculation of accurate %RSD. TSG101, for example, could not be quantified in EVs from either SEC method. Meanwhile, this analyte was reproducibly quantifiable in ExoQuick isolates, exhibiting 14% and 6.9% RSD in isolations and digests, respectively. Similarly, CANX was < LLOQ in qEV70 but could be reproducibly quantified in qEV35 and ExoQuick EVs. Since CANX is relatively enriched in cells compared to EVs, HLM were analysed as a positive control. Normalised to injected protein, CANX signal in EVs compared to HLM was 3.5% and 0.65% in qEV35 and ExoQuick, respectively. Positive EV marker CD9 could be reproducibly quantified across qEV70 and ExoQuick EVs (13% and 18% RSD, respectively). CD81 quantification was also reproducible in ExoQuick isolates but more variable in qEV35 at 37%, which was driven by the lack of quantifiable levels of the analyte in two of the triplicate isolations from one donor. Albumin concentration was 823‐fold and 3.6‐fold higher in ExoQuick and qEV35 isolates, respectively, compared to qEV70, and each exceeded the upper limit of quantification validated for the assay in qEV70 matrix. Relative to an equivalent volume of serum, the amount of albumin in samples isolated by each method was 0.7%, 0.003%, and 0.001%, respectively. Despite significant depletion, albumin remains highly abundant in EV isolates compared to positive EV markers. Albumin quantification was reproducible across isolations in ExoQuick samples, while using SEC, variability was up to 36%. This suggests that while SEC columns, particularly qEV70, are more effective at removing albumin, the samples may be inconsistently affected by free protein contamination. Figure 4 shows the distribution of measured concentrations of analytes across all technical replicates with each isolation method with reference to limits of detection and quantification. Notably, ExoQuick samples are enriched for both CD81 and TSG101 in comparison to SEC samples, while the differences in CD9 are much less pronounced (Figure 4). While qEV70 samples have higher purity (less albumin and calnexin), tetraspanins are more abundant in qEV35 EVs. Differences in marker abundance may be attributed to the columns enriching for vesicles of different size range. Further, recovery of vesicles (particularly CD81‐ and/or CD9‐ positive vesicles) is possibly compromised by decreasing contamination.

**TABLE 8:** Reproducibility of analyte detection in EVs isolated by SEC or precipitation

	Isolations > LOD (%) (*n* = 9)			Equivalent duplicate digests (%) (*n* = 9)		
Analyte	qEV70	qEV35	ExoQuick	qEV70	qEV35	ExoQuick
ALB	100	100	100	100	100	100
CD81	78	100	100	67	100	100
CD9	100	100	100	100	100	100
CANX	100	100	78	100	100	100
TSG101	89	100	100	89	89	100

**TABLE 9:** Reproducibility of analyte quantification in EVs isolated by SEC or precipitation

	Concentration (pmol/ml)			Isolation variability (% RSD)			Digest variability (% RSD)		
Analyte	qEV70	qEV35	ExoQuick	qEV70	qEV35	ExoQuick	qEV70	qEV35	ExoQuick
ALB	34	122	27984	36	30	6.5	2.5	8.1	3.4
CD81	0.25	0.38	8.54	19	37	20	3.8	6.4	20
CD9	0.04	0.16	0.24	18	22	13	20	9.2	6.9
CANX	<LLOQ	0.19	0.54	–	17	5.3	–	14	3.1
TSG101	<LLOQ	<LLOQ	1.55	–	–	14	–	–	6.9

## 4 DISCUSSION

Here we describe the development and validation of a targeted LC/MS‐MS peptide assay for the quantification of markers associated with EVs and non‐vesicle contaminants. A total of five EV ± markers were included in the panel in accordance with the MISEV guidelines (Théry et al., 2018). As stated, samples should be characterised based on the presence of established transmembrane (CD9, CD81) or cytosolic (TSG101) proteins that incorporate in EVs due to roles in biogenesis and trafficking, and the absence or depletion of matrix‐associated contaminants (e.g., serum protein, ALB) and non‐endosomal intracellular compartment proteins (e.g., ER protein, CANX). The described workflow of in‐solution peptide digest coupled with multiplexed panel format provides a high‐throughput quantitative platform for the analysis of clinical samples, requiring only a fraction of that used for immunoblotting. In this regard, LC‐MS/MS represents a valuable approach to streamline the acquisition of data for addressing reporting criteria, while reducing reagent costs, labour, and consumption of human biospecimens, which are often scarce in volume and irreplaceable.

Assay validation and calibration was primarily performed in EVs isolated by qEV70. Numerous studies (Brennan et al., 2020; Gámez‐Valero et al., 2016; Veerman et al., 2021) compare the characteristics and molecular composition of EV isolates produced by various available methods, and increasingly, SEC is selected as the method of choice (Liangsupree et al., 2021; Monguió‐Tortajada et al., 2019; Sidhom et al., 2020). Nonetheless, no single isolation method is considered suitable for all applications and downstream analyses. In the context of proteomic analyses, SEC has been favoured for the relative purity and detection of EV‐associated proteins that can be achieved with marked reproducibility (Monguió‐Tortajada et al., 2019; Vanderboom et al., 2021; Veerman et al., 2021). By comparison, methods that rely on precipitating agents, including ExoQuick, facilitate high recovery but may interfere with vesicle surface composition and co‐isolate large amounts of soluble proteins that mask less abundant EV proteins (Gámez‐Valero et al., 2016; Veerman et al., 2021). Similarly, conventional techniques such as ultracentrifugation suffer poor reproducibility and significant contamination with protein aggregates (Vanderboom et al., 2021).

The concentration range over which the assay was validated comprised eight calibrators to cover concentrations observed in healthy donor serum and beyond to increased levels that may be observed in various disease contexts (Nguyen et al., 2021; Povero et al., 2020; Sehrawat et al., 2021). For all analytes, calibration curves were linear (all *r* ^2^ > 0.99) and the assay exhibited good precision and accuracy. Notably, the concentration range validated for albumin was 100‐fold greater than that for CD9 (Table 3). This aligns with previous findings that highly abundant serum proteins remain at concentrations in SEC isolates many orders of magnitude above that of EV markers (Nguyen et al., 2021; Vanderboom et al., 2021). Blood is a complex matrix from which to extract EVs, given their numbers are predominated by soluble proteins and various types of lipoproteins with similar physical properties. Irrespective, LC‐MS/MS boasts exceptional sensitivity and the present assay exhibited detection limits in the picomolar range. The value of this sensitivity was realised in the context of sample consumption; where for SEC methods, the volume of serum used in immunoblotting was ∼100 μl on average, while only 12.5 μl in equivalent serum volume was injected for LC‐MS/MS analysis (Table S1, Figure S8). Importantly, the latter approach permitted multiplexed analysis without compromising reliability of detection or requiring additional sample.

For immunoblotting, each biological repeat sample was run on a gel once, transferred onto PVDF membrane and re‐probed for CD9, CD81, TSG101, and CANX. The ability to re‐probe the membrane following peroxidase deactivation has been previously demonstrated (Sennepin et al., 2009) and offers a time and sample‐saving solution for multiple detections by western blot. However, in our case, this approach introduced several challenges. EV markers, CD9 and CD81 have a similar molecular weight (MW), with predicted bands at 25 kDa. As PVDF membranes were first probed and imaged for CD81, followed by the stripping, re‐blocking and re‐probing procedure for CD9, it was not possible to confidently assess both. In this case, stripping of the membrane was not successful and CD81 bands were still visible at MW of 25 kDa. Under ideal conditions, enough sample is available to run a gel for one or two markers at different MW. When working with clinical samples of limited sample volume, there is often only enough sample for one immunoblot, presenting a challenge to the fulfilment of MISEV criteria (Théry et al., 2018). In this study, lysed EV samples were run only once using a set volume of starting material, thereby accentuating the limitations of immunoblotting that are overcome by our LC‐MS/MS approach. With respect to quantitation, immunoblotting can successfully determine the presence and relative abundance of analytes between sample types or groups. Densitometric analysis was performed here but was affected by high background in some positive control (HLM) lanes (Figure S6A & D), resulting in an overestimation of protein yield and skewed representation of the data from these lanes. In contrast, the LC‐MS/MS assay could be validated for the absolute quantification of analytes using stable isotope‐labelled peptides spiked into samples at known concentrations.

The degree and composition of contamination are key determinants of signal intensity and reproducibility in LC‐MS/MS analysis. Peptides derived from non‐vesicular material may co‐elute with target EV markers and suppress ionisation; and those exhibiting similar mass to charge ratios may interfere with particular MRM transitions (Liebler & Zimmerman, 2013). The presence of different vesicle populations or contaminants, isolated by different methods, introduces different sources of interference (i.e., lipid or peptide composition) and therefore greater variability in marker detection and quantification. These matrix effects were evident in that the absolute recovery of several analytes was reduced in EV matrix irrespective of isolation method (Table 7).

The reproducibility of the assay was considered within two distinct frameworks defined by the potential applications. Primarily, the EV Marker Panel can be used to demonstrate the presence and absence of EV (±) markers in line with the characterisation and reporting criteria (Théry et al., 2018). Hence, reproducibility was assessed based on analyte response around the defined LOD. As the detection of analytes was highly reproducible across replicate isolations and digests using all methods (Table 8), we demonstrate that the EV Marker Panel is fit‐for‐purpose. Importantly, this can be achieved using only a fraction of the sample that would otherwise be required for conventional methods, such as immunoassays.

In addition to addressing reporting requirements, quantification of EV markers may serve other functions, such as normalisation of EV‐associated biomarker abundance, although such application would require assessment of quantitative reproducibility. While target analyte response is expected to be more variable in samples with greater levels of contamination, here, we found that in ExoQuick samples—which invariably contain large amounts of co‐precipitated serum proteins—EV marker quantification was highly reproducible both in terms of replicate isolations and digests. Of the positive EV markers, CD9 quantification was most consistent across tested isolation methods and may be suitable for the purpose of normalising circulating biomarkers. The choice of isolation method is a key consideration for the analysis of both the biomarker and normaliser; but as seen in ExoQuick samples, certain levels of contamination may not be detrimental, providing acceptable reproducibility of isolation and quantification is achieved (Kreimer et al., 2015). The potential for human error is higher with SEC isolation due to greater hands‐on time. A recent study also found more variability in proteomic profiles using methods that require more time and careful collection of EV‐containing fractions (e.g., qEV70 and OptiPrep density gradient), in comparison to quicker and easier methods (including ExoQuick and ExoEasy) (Veerman et al., 2021). Even so, automation of SEC can mitigate user influence and improve reproducibility (Monguió‐Tortajada et al., 2019). Given the major source of protein in most EV preparations are contaminants, and vesicle recovery and purity differ markedly across isolation strategies (Théry et al., 2018; Useckaite et al., 2021), the decision to normalise LC‐MS/MS analyses to volume rather than total protein content is to avoid normalising to an artefact. For clinical samples, patient groups may have more vesicles and EV protein, so the analysis of less sample compared to controls could diminish the ability to detect important differences. When applied to EVs isolated by SEC or precipitation methods, the assay demonstrated the differences in recovery and purity of EVs from equivalent starting material. As ExoQuick is a high recovery, low purity method, high abundance of EV‐positive markers and albumin contamination were observed. Meanwhile, SEC methods show intermediate vesicle recovery and purity, with most substantial albumin depletion achieved by qEV70. Previous studies have also reported very low abundance or non‐detection of TSG101 in SEC EVs from blood (Brennan et al., 2020; Buschmann et al., 2018; Veerman et al., 2021). The heterogenous presence and relative abundance of tetraspanins and other biogenesis pathway‐related proteins in EVs is influenced by originating cell types (Koliha et al., 2016; Kugeratski et al., 2021). EVs isolated from the circulation generally comprise a large majority of haematopoietic cell‐derived vesicles, especially platelet EVs (Koliha et al., 2016; Kugeratski et al., 2021; Matsumoto et al., 2020; Palviainen et al., 2020). Notably, platelet‐derived EVs have been shown to be devoid of CD81 (Koliha et al., 2016), which may account for the levels of this marker observed in lower recovery isolation methods such as SEC.

Though endogenous CD81 was more abundant in ExoQuick, assay sensitivity was reduced in this matrix. The success of other EV applications such as untargeted profiling studies is dependent on pure sample preparations, since peptides derived from abundant serum proteins will be sampled more frequently than scarcer EV peptides in the MS (Liebler & Zimmerman, 2013). With minimal non‐EV interference, profiling may reveal subtype‐specific or tissue‐specific vesicle markers (Karimi et al., 2018). Ultimately, these techniques performed in pure samples should continue to generate insights into EV biogenesis, functions, and diagnostic or prognostic value, and promote the development of novel affinity tools for the selective isolation of subpopulations to improve the utility of liquid biopsy (Newman et al., 2022; Rodrigues et al., 2021).

To summarise, we have developed and validated a targeted LC‐MS/MS method for the detection and quantification of a panel of positive and negative EV markers in EVs from blood. The described workflow may be applied for the fulfillment of standard characterisation and reporting criteria, described by the MISEV guidelines, or to quantify changes in EV proteins as a biomarker normalisation strategy. We illustrate how our approach overcomes several challenges faced with the use of immunoblotting when working with limited volume of clinical samples, particularly in regard to sensitivity, throughput and quantitation.

## CONFLICT OF INTEREST

All authors declare no conflicts of interest.

## PATIENT CONSENT

Human blood samples were obtained from healthy volunteers following confirmation of informed consent.

## Supporting information

Supporting information.

## References

[jex256-bib-0001] Bowalgaha, K. , Elliot, D. J. , Mackenzie, P. I. , Knights, K. M. , Swedmark, S. , & Miners, J. O. (2005). S‐Naproxen and desmethylnaproxen glucuronidation by human liver microsomes and recombinant human UDP‐glucuronosyltransferases (UGT): Role of UGT2B7 in the elimination of naproxen. British Journal of Clinical Pharmacology, 60, 423–433. 10.1111/j.1365-2125.2005.02446.x 16187975 PMC1884820

[jex256-bib-0002] Brennan, K. , Martin, K. , Fitzgerald, S. P. , O'Sullivan, J. , Wu, Y. , Blanco, A. , Richardson, C. , & Mc Gee, M. M. (2020). A comparison of methods for the isolation and separation of extracellular vesicles from protein and lipid particles in human serum. Scientific Reports, 10, 1039. 10.1038/s41598-020-57497-7 31974468 PMC6978318

[jex256-bib-0003] Buschmann, D. , Kirchner, B. , Hermann, S. , Märte, M. , Wurmser, C. , Brandes, F. , Kotschote, S. , Bonin, M. , Steinlein, O. K. , Pfaffl, M. W. , Schelling, G. , & Reithmair, M. (2018). Evaluation of serum extracellular vesicle isolation methods for profiling miRNAs by next‐generation sequencing. Journal of Extracellular Vesicles, 7, 1481321–1481321. 10.1080/20013078.2018.1481321 29887978 PMC5990937

[jex256-bib-0004] Colella, A. D. , Chegenii, N. , Tea, M. N. , Gibbins, I. L. , Williams, K. A. , & Chataway, T. K. (2012). Comparison of Stain‐Free gels with traditional immunoblot loading control methodology. Analytical Biochemistry, 430, 108–110. 10.1016/j.ab.2012.08.015 22929699

[jex256-bib-0005] Gámez‐Valero, A. , Monguió‐Tortajada, M. , Carreras‐Planella, L. , Franquesa, M. L. , Beyer, K. , & Borràs, F. E. (2016). Size‐exclusion chromatography‐based isolation minimally alters extracellular vesicles’ characteristics compared to precipitating agents. Scientific Reports, 6, 33641. 10.1038/srep33641 27640641 PMC5027519

[jex256-bib-0006] Greening, D. W. , Xu, R. , Gopal, S. K. , Rai, A. , & Simpson, R. J. (2017). Proteomic insights into extracellular vesicle biology—Defining exosomes and shed microvesicles. Expert Review of Proteomics, 14, 69–95. 10.1080/14789450.2017.1260450 27838931

[jex256-bib-0007] Hirsova, P. , Ibrahim, S. H. , Verma, V. K. , Morton, L. A. , Shah, V. H. , Larusso, N. F. , Gores, G. J. , & Malhi, H. (2016). Extracellular vesicles in liver pathobiology: Small particles with big impact. Hepatology, 64, 2219–2233. 10.1002/hep.28814 27628960 PMC5115968

[jex256-bib-0008] Hofmann, L. , Ludwig, S. , Schuler, P. J. , Hoffmann, T. K. , Brunner, C. , & Theodoraki, M‐N. (2020). The potential of CD16 on plasma‐derived exosomes as a liquid biomarker in head and neck cancer. International Journal of Molecular Sciences, 21, 3739. 10.3390/ijms21113739 32466374 PMC7312379

[jex256-bib-0009] Karimi, N. , Cvjetkovic, A. , Jang, S. C. , Crescitelli, R. , Hosseinpour Feizi, M. A. , Nieuwland, R. , Lötvall, J. , & Lässer, C. (2018). Detailed analysis of the plasma extracellular vesicle proteome after separation from lipoproteins. Cellular and Molecular Life Sciences, 75, 2873–2886. 10.1007/s00018-018-2773-4 29441425 PMC6021463

[jex256-bib-0010] Koliha, N. , Wiencek, Y. , Heider, U. , JÃ¼Ngst, C. , Kladt, N. , Krauthäuser, S. , Johnston, I. C. D. , Bosio, A. , Schauss, A. , & Wild, S. (2016). A novel multiplex bead‐based platform highlights the diversity of extracellular vesicles. Journal of Extracellular Vesicles, 5, 29975. 10.3402/jev.v5.29975 26901056 PMC4762227

[jex256-bib-0011] Kowal, J. , Arras, G. , Colombo, M. , Jouve, M. , Morath, J. P. , Primdal‐Bengtson, B. , Dingli, F. , Loew, D. , Tkach, M. , & Théry, C. (2016). Proteomic comparison defines novel markers to characterize heterogeneous populations of extracellular vesicle subtypes. Proceedings of the National Academy of Sciences, 113, E968–E977. 10.1073/pnas.1521230113 PMC4776515 26858453

[jex256-bib-0012] Kreimer, S. , Belov, A. M. , Ghiran, I. , Murthy, S. K. , Frank, D. A. , & Ivanov, A. R. (2015). Mass‐spectrometry‐based molecular characterization of extracellular vesicles: Lipidomics and proteomics. Journal of Proteome Research, 14, 2367–2384. 10.1021/pr501279t 25927954

[jex256-bib-0013] Kugeratski, F. G. , Hodge, K. , Lilla, S. , Mcandrews, K. M. , Zhou, X. , Hwang, R. F. , Zanivan, S. , & Kalluri, R. (2021). Quantitative proteomics identifies the core proteome of exosomes with syntenin‐1 as the highest abundant protein and a putative universal biomarker. Nature Cell Biology, 23, 631–641. 10.1038/s41556-021-00693-y 34108659 PMC9290189

[jex256-bib-0014] Liangsupree, T. , Multia, E. , & Riekkola, M‐L. (2021). Modern isolation and separation techniques for extracellular vesicles. Journal of Chromatography A, 1636, 461773. 10.1016/j.chroma.2020.461773 33316564

[jex256-bib-0015] Liebler, D. C. , & Zimmerman, L. J. (2013). Targeted quantitation of proteins by mass spectrometry. Biochemistry, 52, 3797–3806. 10.1021/bi400110b 23517332 PMC3674507

[jex256-bib-0016] Matsumoto, A. , Takahashi, Y. , Chang, H. Y. , Wu, Y. W. , Yamamoto, A. , Ishihama, Y. , & Takakura, Y. (2020). Blood concentrations of small extracellular vesicles are determined by a balance between abundant secretion and rapid clearance. Journal of Extracellular Vesicles, 9, 1696517. 10.1080/20013078.2019.1696517 31807238 PMC6882433

[jex256-bib-0017] Monguió‐Tortajada, M. , Gálvez‐Montón, C. , Bayes‐Genis, A. , Roura, S. , & Borràs, F. E. (2019). Extracellular vesicle isolation methods: Rising impact of size‐exclusion chromatography. Cellular and Molecular Life Sciences, 76, 2369–2382. 10.1007/s00018-019-03071-y 30891621 PMC11105396

[jex256-bib-0018] Newman, L. A. , Fahmy, A. , Sorich, M. J. , Best, O. G. , Rowland, A. , & Useckaite, Z. (2021). Importance of between and within subject variability in extracellular vesicle abundance and cargo when performing biomarker analyses. Cells, 10, 485. 33668220 10.3390/cells10030485 PMC7996254

[jex256-bib-0019] Newman, L. A. , Sorich, M. J. , & Rowland, A. (2020). Role of extracellular vesicles in the pathophysiology, diagnosis and tracking of non‐alcoholic fatty liver disease. Journal of Clinical Medicine, 9, 2032. 32610455 10.3390/jcm9072032 PMC7409057

[jex256-bib-0020] Newman, L. A. , Useckaite, Z. , Johnson, J. , Sorich, M. J. , Hopkins, A. M. , & Rowland, A. (2022). Selective isolation of liver‐derived extracellular vesicles redefines performance of miRNA biomarkers for non‐alcoholic fatty liver disease. Biomedicines, 10, 195. 35052873 10.3390/biomedicines10010195 PMC8773667

[jex256-bib-0021] Nguyen, A. , Wang, T. , & Turko, I. V. (2021). Quantitative proteomic analysis for evaluating affinity isolation of extracellular vesicles. Journal of Proteomics, 249, 104359. 10.1016/j.jprot.2021.104359 34454076

[jex256-bib-0022] Palviainen, M. , Saraswat, M. , Varga, Z. , Kitka, D. , Neuvonen, M. , Puhka, M. , Joenväärä, S. , Renkonen, R. , Nieuwland, R. , Takatalo, M. , & Siljander, P. R. M. (2020). Extracellular vesicles from human plasma and serum are carriers of extravesicular cargo—Implications for biomarker discovery. PLoS One, 15, e0236439. 10.1371/journal.pone.0236439 32813744 PMC7446890

[jex256-bib-0023] Park, J. , Go, E‐B. , Oh, J. S. , Lee, J. K. , & Lee, S‐Y. (2021). Multiple‐cycle polymeric extracellular vesicle precipitation and its evaluation by targeted mass spectrometry. International Journal of Molecular Sciences, 22, 4311. 10.3390/ijms22094311 33919183 PMC8122279

[jex256-bib-0024] Povero, D. , Yamashita, H. , Ren, W. , Subramanian, M. G. , Myers, R. P. , Eguchi, A. , Simonetto, D. A. , Goodman, Z. D. , Harrison, S. A. , Sanyal, A. J. , Bosch, J. , & Feldstein, A. E. (2020). Characterization and proteome of circulating extracellular vesicles as potential biomarkers for NASH. Hepatology Communications, 4, 1263–1278. 10.1002/hep4.1556 32923831 PMC7471415

[jex256-bib-0025] Rodrigues, A. D. , van Dyk, M. , Sorich, M. J. , Fahmy, A. , Useckaite, Z. , Newman, L. A. , Kapetas, A. J. , Mounzer, R. , Wood, L. S. , Johnson, J. G. , & Rowland, A. (2021). Exploring the use of serum‐derived small extracellular vesicles as liquid biopsy to study the induction of hepatic cytochromes P450 and organic anion transporting polypeptides. Clinical Pharmacology & Therapeutics, 110, 248–258. 10.1002/cpt.2244 33792897

[jex256-bib-0026] Rosa‐Fernandes, L. , Rocha, V. B. , Carregari, V. C. , Urbani, A. , & Palmisano, G. (2017). A perspective on extracellular vesicles proteomics. Frontiers in Chemistry, 5, 102–102. 10.3389/fchem.2017.00102 29209607 PMC5702361

[jex256-bib-0027] Schroeder, J. C. , Puntigam, L. , Hofmann, L. , Jeske, S. S. , Beccard, I. J. , Doescher, J. , Laban, S. , Hoffmann, T. K. , Brunner, C. , Theodoraki, M‐N. , & Schuler, P. J. (2020). Circulating exosomes inhibit B cell proliferation and activity. Cancers (Basel), 12, 2110. 10.3390/cancers12082110 32751214 PMC7464446

[jex256-bib-0028] Sehrawat, T. S. , Arab, J. P. , Liu, M. , Amrollahi, P. , Wan, M. , Fan, J. , Nakao, Y. , Pose, E. , Navarro‐Corcuera, A. , Dasgupta, D. , Liao, C. Y. , He, L. , Mauer, A. S. , Avitabile, E. , Ventura‐Cots, M. , Bataller, R. A. , Sanyal, A. J. , Chalasani, N. P. , Heimbach, J. K. , …, Malhi, H. (2021). Circulating extracellular vesicles carrying sphingolipid cargo for the diagnosis and dynamic risk profiling of alcoholic hepatitis. Hepatology, 73, 571–585. 10.1002/hep.31256 32246544 PMC7541595

[jex256-bib-0029] Sennepin, A. D. , Charpentier, S. , Normand, T. , Sarré, C. , Legrand, A. , & Mollet, L. M. (2009). Multiple reprobing of Western blots after inactivation of peroxidase activity by its substrate, hydrogen peroxide. Analytical Biochemistry, 393, 129–131. 10.1016/j.ab.2009.06.004 19523435

[jex256-bib-0030] Shao, H. , Im, H. , Castro, C. M. , Breakefield, X. , Weissleder, R. , & Lee, H. (2018). New technologies for analysis of extracellular vesicles. Chemical Reviews, 118, 1917–1950. 10.1021/acs.chemrev.7b00534 29384376 PMC6029891

[jex256-bib-0031] Sidhom, K. , Obi, P. O. , & Saleem, A. (2020). A review of exosomal isolation methods: Is size exclusion chromatography the best option? International Journal of Molecular Sciences, 21, 6466. 10.3390/ijms21186466 32899828 PMC7556044

[jex256-bib-0032] Théry, C. , Witwer, K. W. , Aikawa, E. , Alcaraz, M. J. , Anderson, J. D. , Andriantsitohaina, R. , Antoniou, A. , Arab, T. , Archer, F. , Atkin‐Smith, G. K. , Ayre, D. C. , Bach, J‐M. , Bachurski, D. , Baharvand, H. , Balaj, L. , Baldacchino, S. , Bauer, N. N. , Baxter, A. A. , Bebawy, M. , …, Zuba‐Surma, E. K. (2018). Minimal information for studies of extracellular vesicles 2018 (MISEV2018): A position statement of the International Society for Extracellular Vesicles and update of the MISEV2014 guidelines. Journal of Extracellular Vesicles, 7, 1535750. 10.1080/20013078.2018.1535750 30637094 PMC6322352

[jex256-bib-0033] Useckaite, Z. , Mukhopadhya, A. , Moran, B. , & O'Driscoll, L. (2020). Extracellular vesicles report on the MET status of their cells of origin regardless of the method used for their isolation. Scientific Reports, 10, 19020. 10.1038/s41598-020-75817-9 33149187 PMC7642384

[jex256-bib-0034] Useckaite, Z. , Rodrigues, A. D. , Hopkins, A. M. , Newman, L. A. , Johnson, J. , Sorich, M. J. , & Rowland, A. (2021). Role of extracellular vesicle derived biomarkers in drug metabolism and disposition. Drug Metabolism and Disposition, 49, 961–971. 10.1124/dmd.121.000411 34353847

[jex256-bib-0035] Vanderboom, P. M. , Dasari, S. , Ruegsegger, G. N. , Pataky, M. W. , Lucien, F. , Heppelmann, C. J. , Lanza, I. R. , & Nair, K. S. (2021). A size‐exclusion‐based approach for purifying extracellular vesicles from human plasma. Cell Reports Methods, 1, 100055. 10.1016/j.crmeth.2021.100055 34355211 PMC8336930

[jex256-bib-0036] Van Deun, J. , Mestdagh, P. , Agostinis, P. , Akay, Ö. , Anand, S. , Anckaert, J. , Martinez, Z. A. , Baetens, T. , Beghein, E. , Bertier, L. , Berx, G. , Boere, J. , Boukouris, S. , Bremer, M. , Buschmann, D. , Byrd, J. B. , Casert, C. , Cheng, L. , Cmoch, A. , …, Hendrix, A. (2017). EV‐TRACK: Transparent reporting and centralizing knowledge in extracellular vesicle research. Nature Methods, 14, 228–232. 10.1038/nmeth.4185 28245209

[jex256-bib-0037] Veerman, R. E. , Teeuwen, L. , Czarnewski, P. , Güclüler Akpinar, G. , Sandberg, A. , Cao, X. , Pernemalm, M. , Orre, L. M. , Gabrielsson, S. , & Eldh, M. (2021). Molecular evaluation of five different isolation methods for extracellular vesicles reveals different clinical applicability and subcellular origin. Journal of Extracellular Vesicles, 10, e12128. 10.1002/jev2.12128 34322205 PMC8298890

[jex256-bib-0038] Wang, T. , Anderson, K. W. , & Turko, I. V. (2017). Assessment of extracellular vesicles purity using proteomic standards. Analytical Chemistry, 89, 11070–11075. 10.1021/acs.analchem.7b03119 28949504 PMC5896757

[jex256-bib-0039] Webber, J. , & Clayton, A. (2013). How pure are your vesicles? Journal of Extracellular Vesicles, 2, 19861. 10.3402/jev.v2i0.19861 PMC3760653 24009896

[jex256-bib-0040] Witwer, K. W. , Goberdhan, D. C. , O'driscoll, L. , Théry, C. , Welsh, J. A. , Blenkiron, C. , Buzás, E. I. , Di Vizio, D. , Erdbrügger, U. , Falcón‐Pérez, J. M. , Fu, Q. L. , Hill, A. F. , Lenassi, M. , Lötvall, J. , Nieuwland, R. , Ochiya, T. , Rome, S. , Sahoo, S. , & Zheng, L. (2021). Updating MISEV: Evolving the minimal requirements for studies of extracellular vesicles. Journal of Extracellular Vesicles, 10, e12182. 10.1002/jev2.12182 34953156 PMC8710080

[jex256-bib-0041] Zhang, L. , Parot, J. , Hackley, V. A. , & Turko, I. V. (2020). Quantitative proteomic analysis of biogenesis‐based classification for extracellular vesicles. Proteomes, 8, 33. 10.3390/proteomes8040033 33171920 PMC7709127

