# Supplementary material for: Addressing MISEV guidance using targeted LC‐MS/MS: A method for the detection and quantification of extracellular vesicle‐enriched and contaminant protein markers from blood
Source: J Extracell Biol. 2022 Sep 5;1(9):e56. doi: 10.1002/jex2.56 (PMC11080780; doi:10.1002/jex2.56)
Supplement: Supplementary file 1 — Supporting information. [file JEX2-1-e56-s001.pdf]

Supporting Information

Addressing MISEV Guidance Using Targeted LC-MS/MS: A Method for the Detection and Quantification of Extracellular Vesicle-Enriched and Contaminant Protein Markers from Blood

Lauren A. Newman[1], Zivile Useckaite[1], and Andrew Rowland[1]

[1] College of Medicine and Public Health, Flinders University, Adelaide, South Australia
Corresponding author email: andrew.rowland@flinders.edu.au

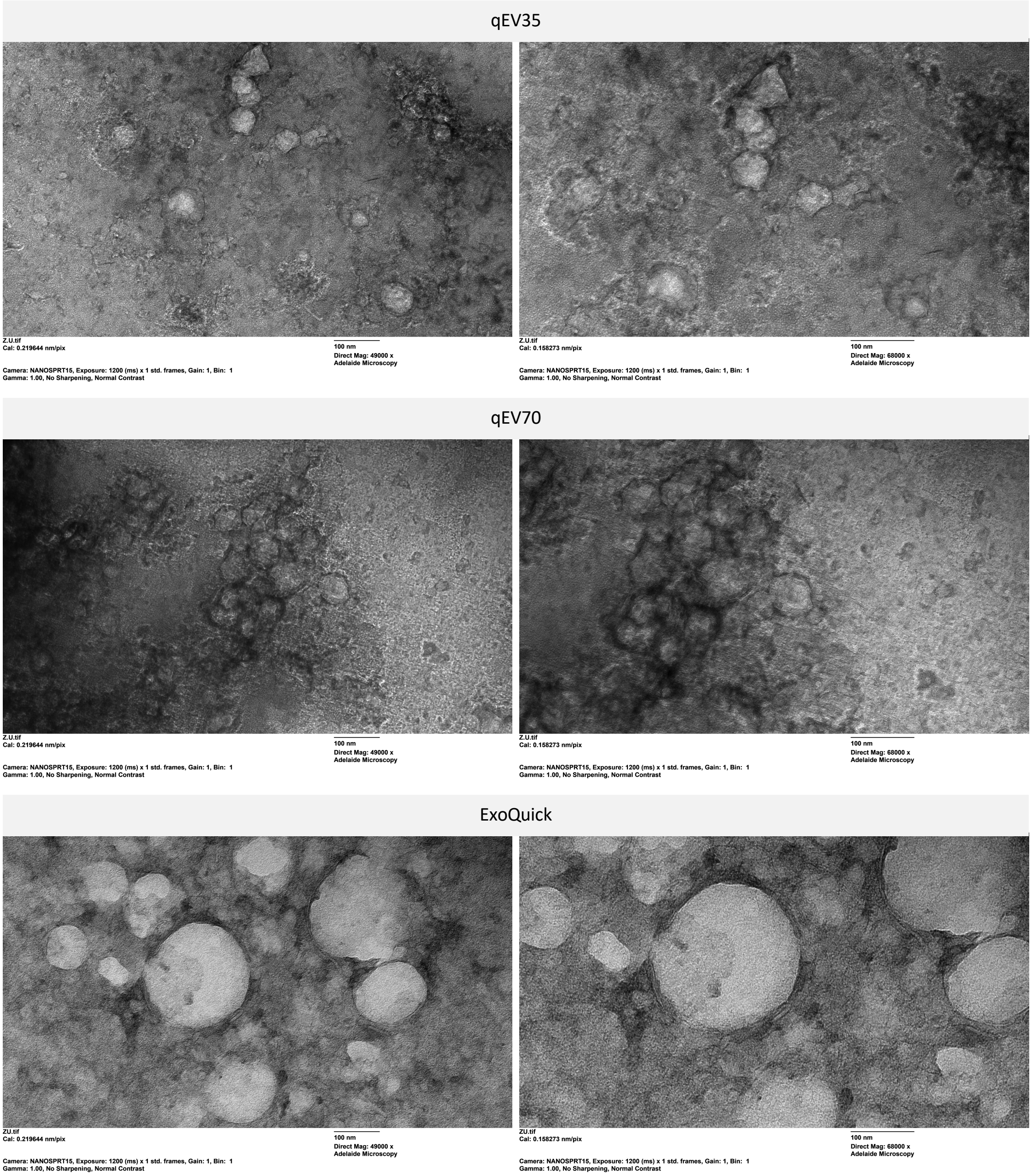

Figure S1. Unedited TEM images at 49,000 x magnification (left panels) and 68,000 x magnification (right panels).

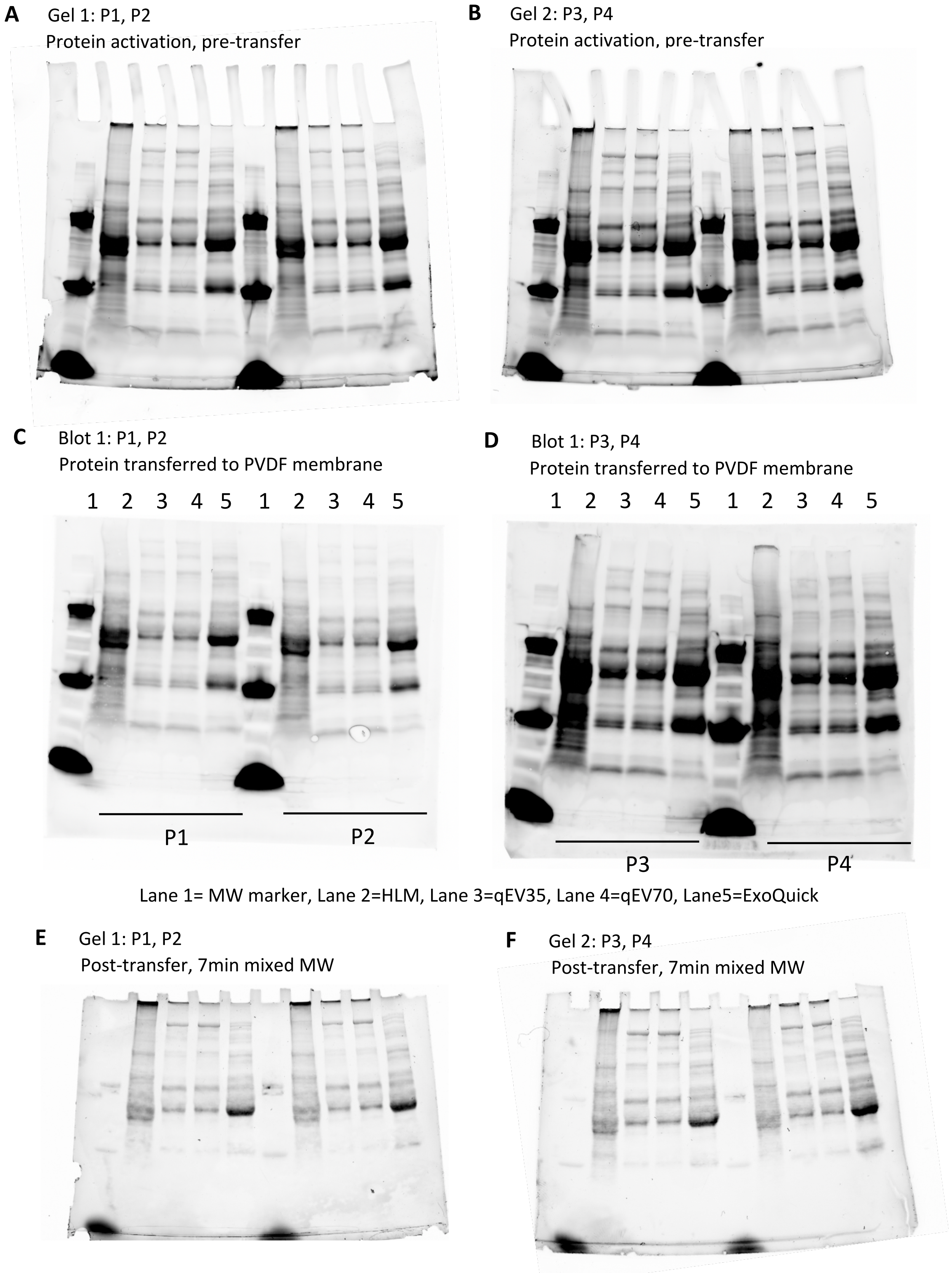

Figure S2. Full images of immunoblot gels showing total protein activation pre-transfer **(A-B)** and post-transfer **(E-F)** onto PVDF membranes **(C-D)**. No stain, 35µg of lysed HLM or EV protein loaded to each well.

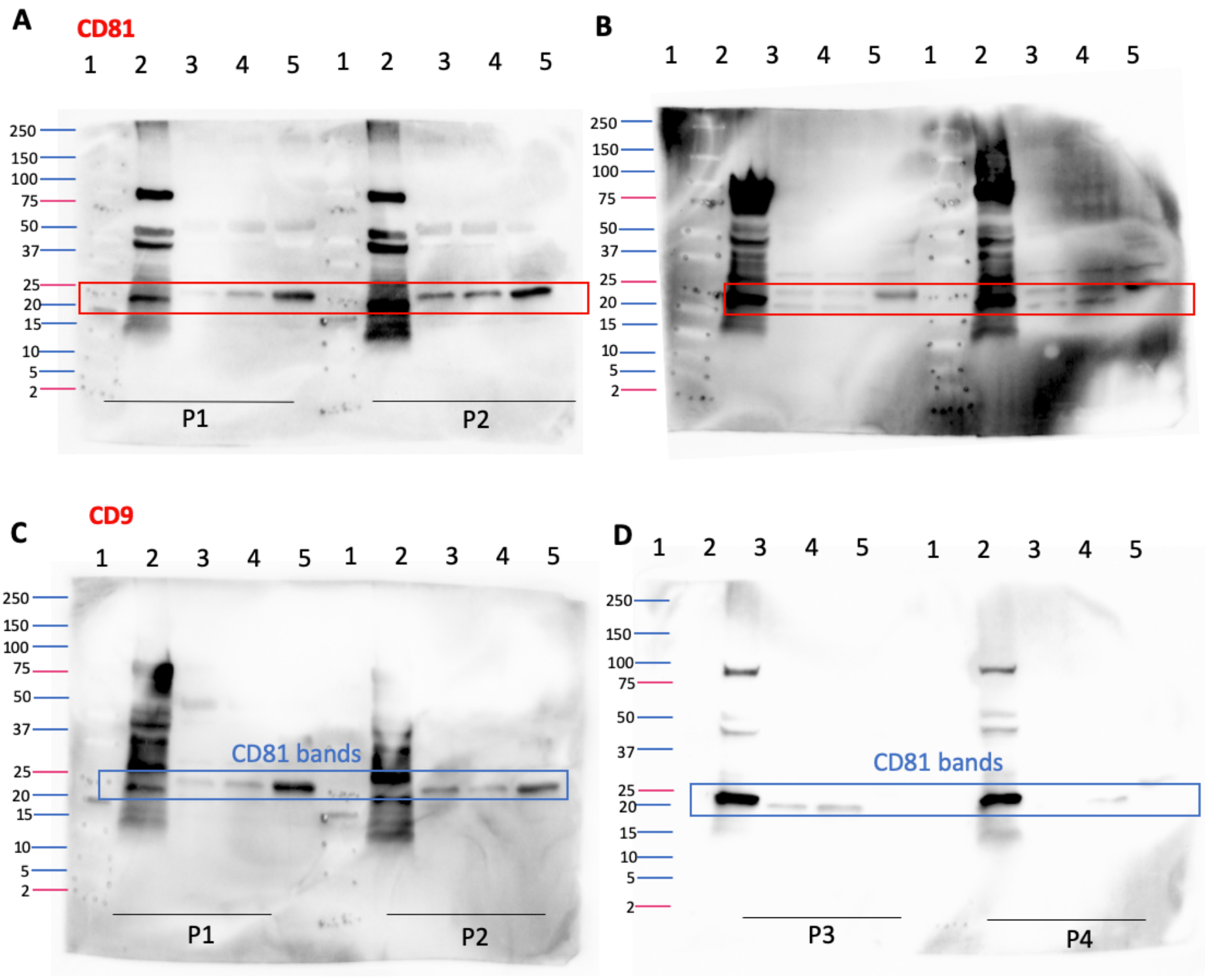

Figure S3. Full images of immunoblots the expression of EV-associated tetraspanins: CD81 **(A-B)** and CD9 **(C-D)** on PVDF. No CD9 band observed, unsuccessful stripping of CD81 antibody. Auto-exposure, 35µg of lysed HLM or EV protein loaded to each well. Lane 1= MW marker, Lane 2=HLM, Lane 3=qEV35, Lane 4=qEV70, Lane 5=ExoQuick

CD81 predicted band 25kDA, observed band 22kDa
CD9 predicted band 25kDa, reported 22kDa on manufacturers website. Not observed.

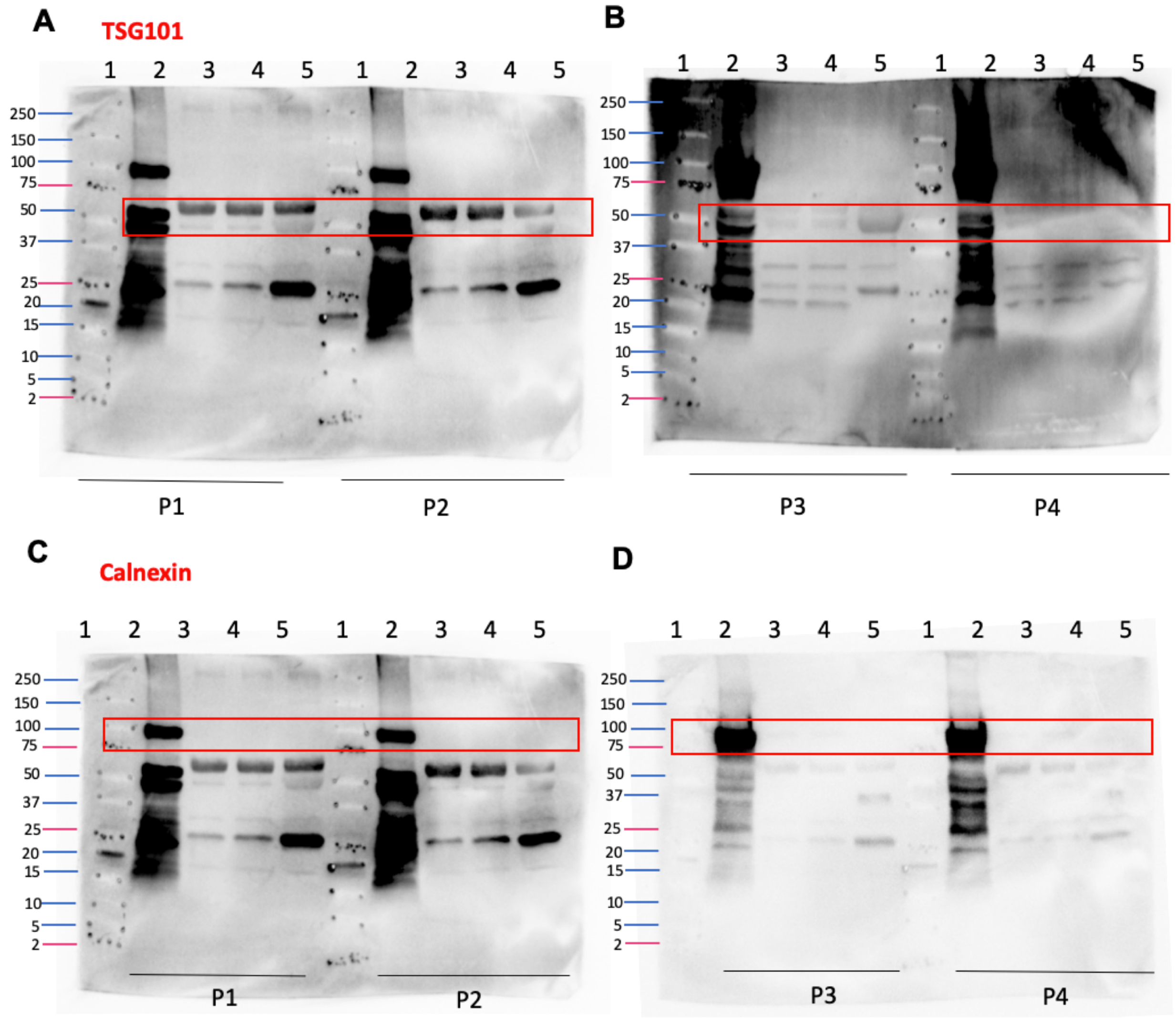

Figure S4. Full images of immunoblots the expression of EV-associated TSG101 (**A-B)** and **(A-B)** and ER marker Calnexin **(C-D)** on PVDF. Auto exposure, 35µg of lysed HLM or EV protein loaded to each well. Lane 1= MW marker, Lane 2=HLM, Lane 3=qEV35, Lane 4=qEV70, Lane5=ExoQuick.

TSG101 predicted band ~50kDa, observed double band at 48-55kDa
Calnexin 5 predicted band at ~90kDa, observed at ~95kDa

Albumin in Serum vs EV lysates

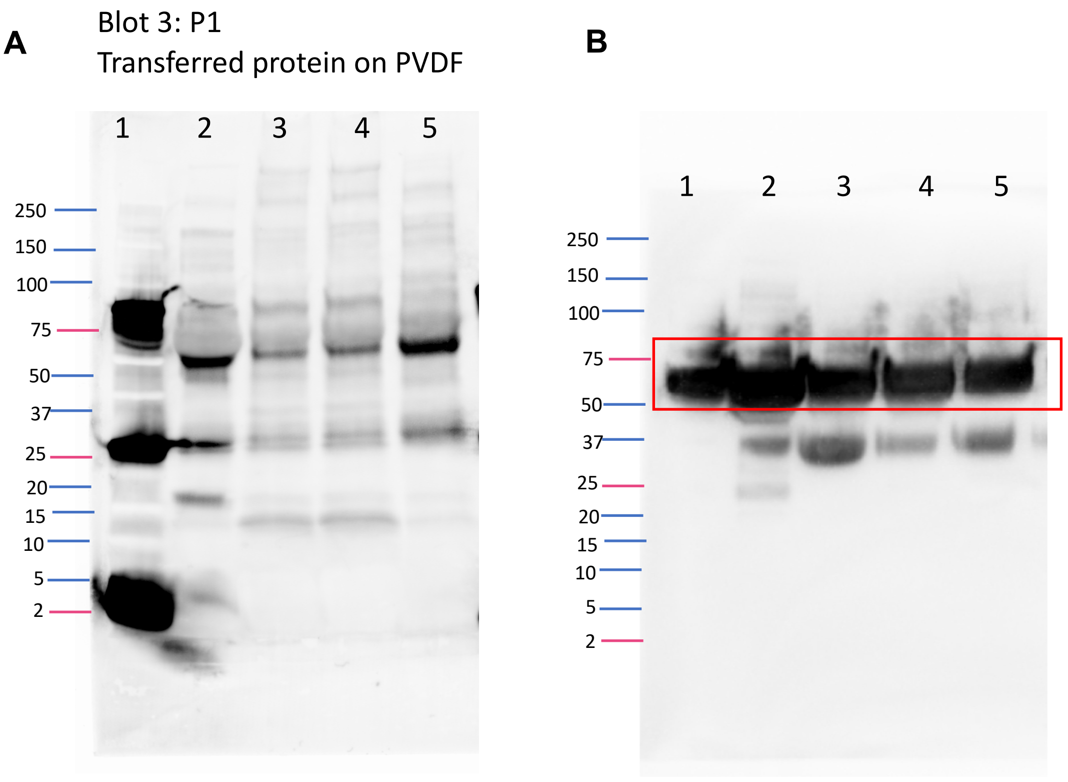

Figure S5. (A) Full images protein on PVDF/stain-free; **(B)** Full image of immunoblot showing Albumin expression at ~ 70kDa. Lane 1=MW marker, Lane 2=HLM, Lane 3=qEV35, Lane 4=qEV70, Lane5=ExoQuick.; Albumin protein leaked from Lane 2 into Lane 1, therefore, Lane 1 and Lane 2 are considered to be one well in terms of Albumin protein expression. EV protein and matched serum samples from P1 were used; 35µg of lysed serum or lysed EV protein loaded to each well.

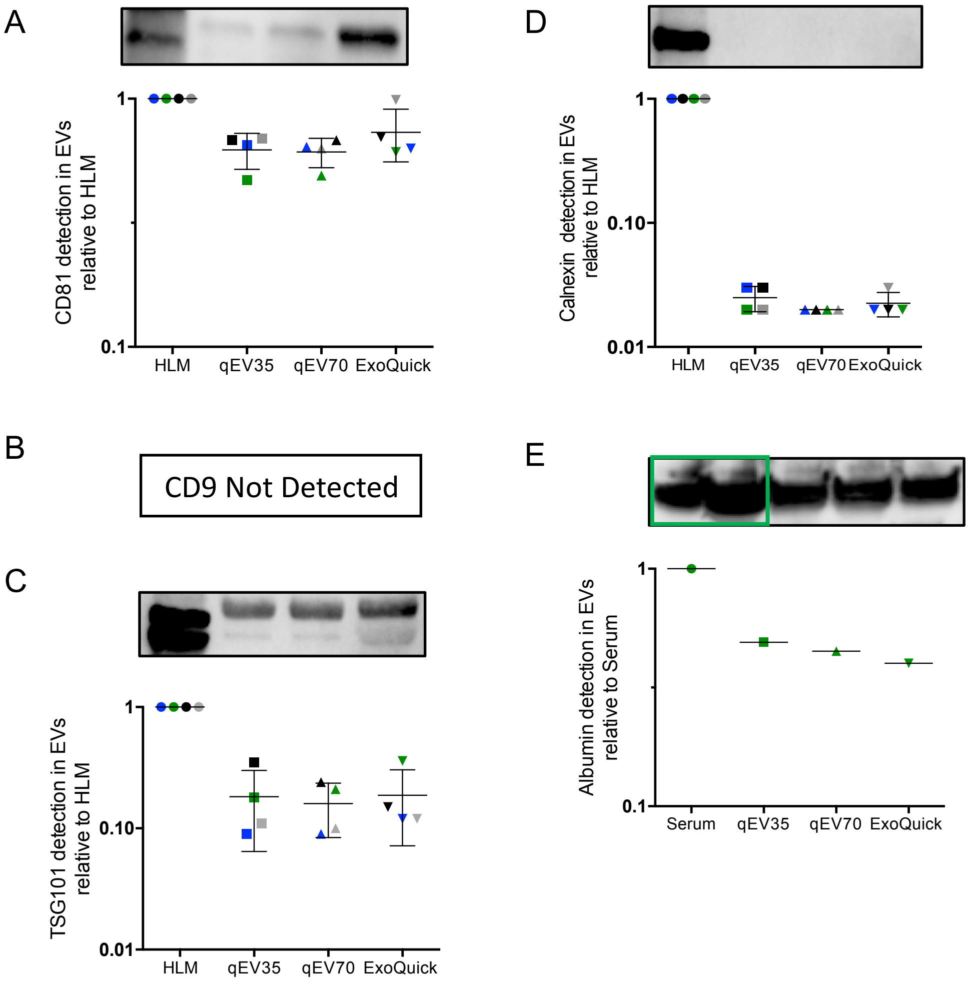

Figure S6. Relative protein quantification by ImageJ. ImageJ obtained values for non-EV controls (HLM) **(A-D)** and serum **(E)** were set to one and values obtained for corresponding EV lanes were expressed as fold change relative to non-EV control. The main focus was to compare protein expression between EVs obtained by three isolation methods, however, non-EV controls were included.

Figure S7. Representative chromatograms in Skyline. Stable isotope labelled peptides [ALB: 80 pmol/mL, CD81: 8 pmol/mL, CD9: 0.8 pmol/mL, CANX: 1.6 pmol/mL, TSG101: 1.6 pmol/mL] spiked into EV digests (qEV70) from healthy human serum.

Albumin

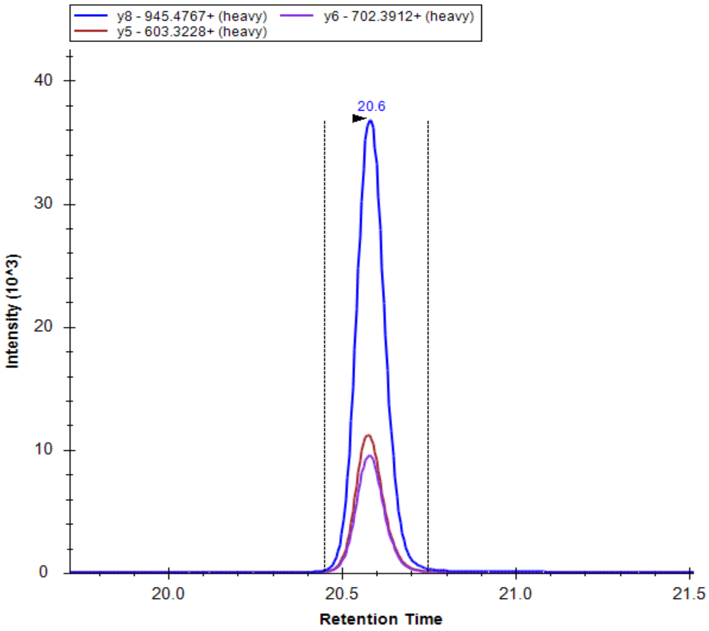

CD81

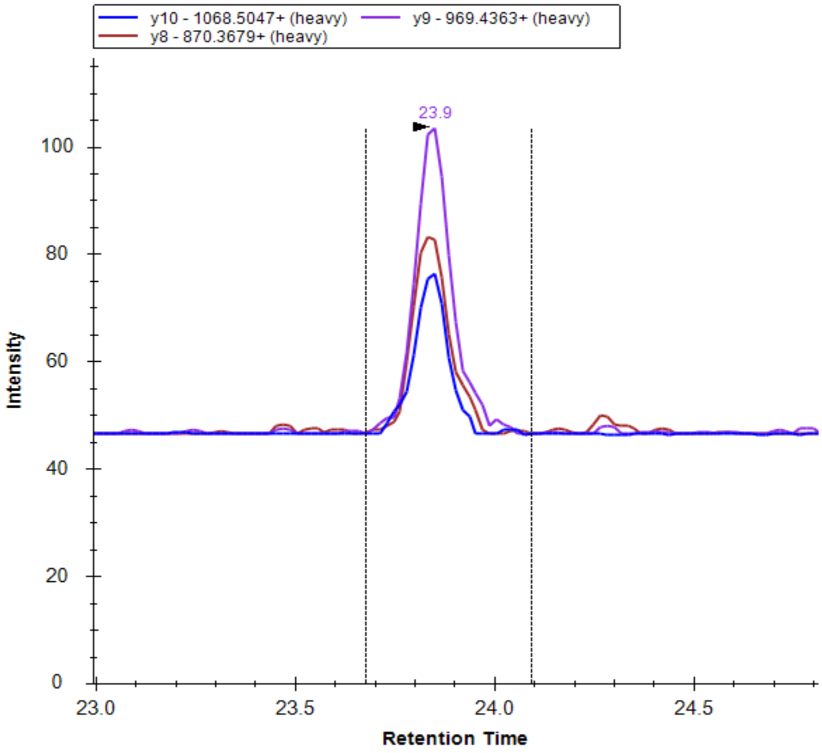

CD9

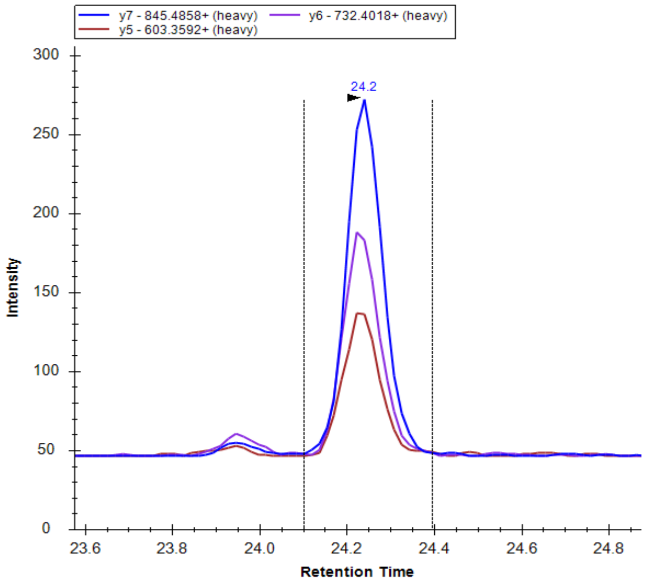

CANX

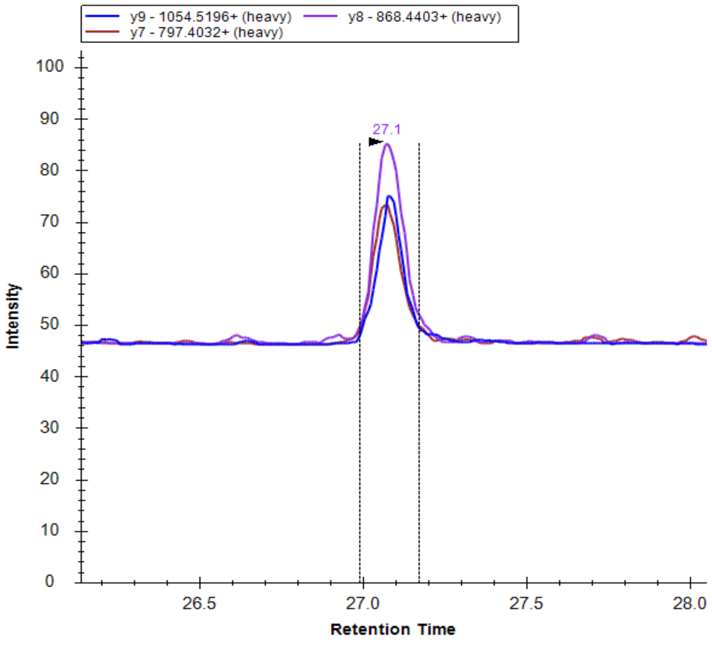

TSG101

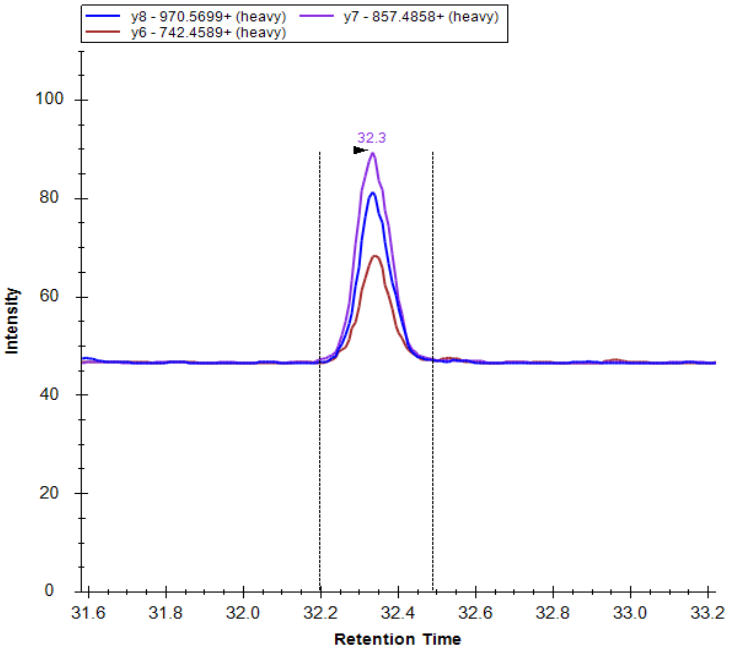

Figure S8. Comparison of sample consumption for immunoblots and LC-MS/MS injections in EV marker analytical workflow.

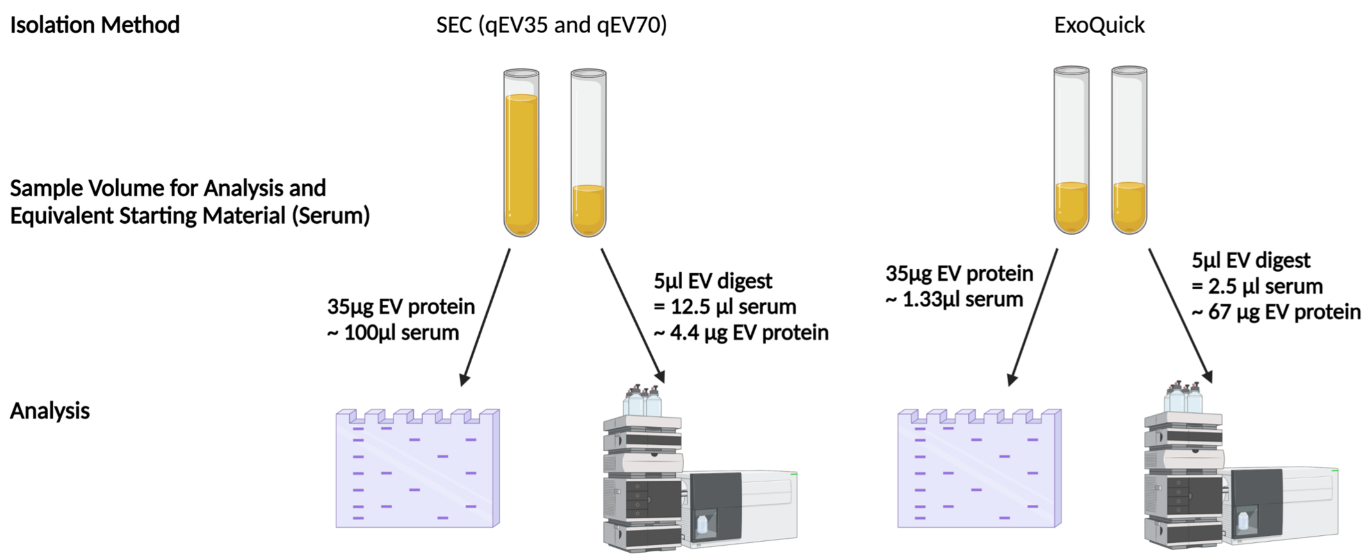

Table S1. Protein concentration of EV samples, measured by micro BCA, and comparison of sample consumptions for immunoblots and LC-MS/MS injections. Immunoblot protein loading = 35 µg. LC-MS/MS injection volume = 5µl.

			Immunoblots		LC-MS/MS	
Method	Donor	Protein Concentration (µg/µl)	Volume EVs Loaded (µl)	Equivalent Serum Volume (µl)	Protein per Injection (µg)	Equivalent Serum Volume (µl)
qEV35	1	1.71	20.4	102.1	4.28	12.5
	2	1.68	20.8	104.2	4.20	
	3	1.54	22.7	113.6	3.85	
	4	1.41	24.8	124.1	3.53	
qEV70	1	1.92	18.2	91.1	4.80	12.5
	2	1.67	20.9	104.7	4.18	
	3	2.40	14.6	72.8	6.00	
	4	1.85	18.9	94.6	4.63	
ExoQuick	1	139.27	0.25	1.26	69.64	2.5
	2	116.42	0.30	1.50	58.21	
	3	113.22	0.31	1.55	56.61	
	4	168.33	0.21	1.04	84.17	

Table S2. Detection of EV markers in serum and plasma EVs from healthy donors and patients with NAFLD using qEV70 and ExoQuick.

**Analyte**	**qEV70 EVs > LOD (%)**			**ExoQuick EVs > LOD (%)**	
	Healthy Serum (n=9)	Healthy Plasma (n=5)	NAFLD Plasma (n=4)	Healthy Serum (n=9)	NAFLD Serum (n=5)
**ALB**	100	100	100	100	100
**CD81**	78	80	100	100	100
**CD9**	100	100	100	100	100
**CANX**	100	20	80	78	100
**TSG101**	89	80	100	100	100
